# Organokines in Rheumatoid Arthritis: A Critical Review

**DOI:** 10.3390/ijms23116193

**Published:** 2022-05-31

**Authors:** Lucas Fornari Laurindo, Mariana Canevari de Maio, Sandra Maria Barbalho, Elen Landgraf Guiguer, Adriano Cressoni Araújo, Ricardo de Alvares Goulart, Uri Adrian Prync Flato, Edgar Baldi Júnior, Cláudia Rucco Penteado Detregiachi, Jesselina Francisco dos Santos Haber, Patrícia C. Santos Bueno, Raul S. J. Girio, Rachel Gomes Eleutério, Marcelo Dib Bechara

**Affiliations:** 1 Department of Biochemistry and Pharmacology, School of Medicine, University of Marília (UNIMAR), Avenida Hygino Muzzy Filho, 1001, Marília, São Paulo 17525-902, Brazil; lucasffffor@gmail.com (L.F.L.); elguiguer@gmail.com (E.L.G.); adrianocressoniaraujo@yahoo.com.br (A.C.A.); rgoulart@unimar.br (R.d.A.G.); uriflato@gmail.com (U.A.P.F.); reumatoedgar@hotmail.com (E.B.J.); haber.jesselina@gmail.com (J.F.d.S.H.); patriciabueno@unimar.br (P.C.S.B.); dib.marcelo1@gmail.com (M.D.B.); 2 Department of Biochemistry and Pharmacology, School of Medicine, Faculty of Medicine of Marília (FAMEMA), Avenida Monte Carmelo, 800, Marília, São Paulo 17519-030, Brazil; mcanevaridemaio@gmail.com; 3 Postgraduate Program in Structural and Functional Interactions in Rehabilitation, University of Marília (UNIMAR), Avenida Hygino Muzzy Filho, 1001, Marília, São Paulo 17525-902, Brazil; claudia.detregiachi@unimar.br; 4 Department of Biochemistry, School of Food and Technology of Marilia (FATEC), Avenida Castro Alves, 62, Marília, São Paulo 17500-000, Brazil; 5 Department of Animal Sciences, School of Veterinary Medicine, University of Marília (UNIMAR), Avenida Hygino Muzzy Filho, 1001, Marília, São Paulo 17525-902, Brazil; rgirio@unimar.br; 6 Department of Biochemistry, School of Dentistry, University of Marília (UNIMAR), Avenida Hygino Muzzy Filho, 1001, Marília, São Paulo 17525-902, Brazil; rachelgomes@hotmail.com

**Keywords:** organokines, myokines, osteokines, adipokines, hepatokines, rheumatoid arthritis, inflammation, oxidative stress, crosstalk, rheumatology

## Abstract

Rheumatoid arthritis (RA) is a systemic autoimmune disease that primarily affects the joints. Organokines can produce beneficial or harmful effects in this condition. Among RA patients, organokines have been associated with increased inflammation and cartilage degradation due to augmented cytokines and metalloproteinases production, respectively. This study aimed to perform a review to investigate the role of adipokines, osteokines, myokines, and hepatokines on RA progression. *PubMed, Embase, Google Scholar,* and *Cochrane* were searched, and 18 studies were selected, comprising more than 17,000 RA patients. Changes in the pattern of organokines secretion were identified, and these could directly or indirectly contribute to aggravating RA, promoting articular alterations, and predicting the disease activity. In addition, organokines have been implicated in higher radiographic damage, immune dysregulation, and angiogenesis. These can also act as RA potent regulators of cells proliferation, differentiation, and apoptosis, controlling osteoclasts, chondrocytes, and fibroblasts as well as immune cells chemotaxis to RA sites. Although much is already known, much more is still unknown, principally about the roles of organokines in the occurrence of RA extra-articular manifestations.

## 1. Introduction

Rheumatoid arthritis (RA) is a chronic systemic and autoimmune disease that affects approximately 1% of the world’s population. Being characterized mainly by persistent articular inflammation, this condition affects the synovial membranes of the joints, leading to joint destruction, loss of functions, and osteoarticular disabilities. In the disease’s progression, bone and cartilage are destroyed, which brings deformities to the patients [1,2,3]. Although RA is prevalent worldwide, its incidence is higher among women when compared to men, with an incident ratio of about two or three women to one man, respectively.

The physiopathology of RA is still not fully understood. However, many cells have been implicated in its development. In RA, the joint damage is driven principally due to the activity of proliferative synovial tissue fibroblasts, which are accompanied by neutrophils, monocytes, and T and B lymphocytes trafficking into the articular synovium. These cells are mainly pro-inflammatory, secreting many pro-inflammatory cytokines into the articular cavities [1,2].

Besides inflammation, oxidative stress (OS) also plays an essential role in the pathogenesis and progress of RA impairments. The excessive production of free radicals causes the oxidation of many different molecules in the human body, including articular. These events seem to be positive and extensively associated with augmented inflammation and accelerated joint destruction [3,4]. Due to its complex systemic definition, RA can also be associated with extra-articular manifestations, such as cardiologic, hepatic, pulmonary, digestive, ocular, dermatological, and neurological [5].

In the molecular context, organokines (myokines, osteokines, hepatokines, and adipokines) have been increasingly investigated in the pathophysiology of many diseases, such as insulin resistance (IR), dementia, non-alcoholic fatty liver disease, and cardiovascular affections. They are mainly adipokines, myokines, hepatokines, and osteokines, which are produced by adipose tissue, skeletal muscle, liver, and bones, respectively. Organokines can have beneficial or harmful effects on the human body besides performing crosstalk among different organs. Acting through endocrine, autocrine, or paracrine pathways can evidence inflammatory and oxidative stimuli [6,7]. Recently, organokines have shown an important role in the rheumatological field, inclusive of RA biomarkers.

In RA disease, organokines have been shown to promote inflammation or augment cartilage degradation by increasing pro-inflammatory cytokine production and metalloproteinases (MMPs) secretion, respectively. In turn, these molecules can associate with more serious radiographic damage among RA patients and immune dysregulation, combining T-cells differentiation and angiogenesis stimuli. Among many other actions, organokines are associated with the RA disease progression, and the roles of these molecules in RA and their possible cross-talks must become clearer [8,9,10].

Considering the aforementioned information and that organokines can act combined to improve health or cause disease [11] and can play a role in RA, this study aimed to review the molecular and biochemical aspects of myokines, adipokines, hepatokines, and osteokines and their relationship with RA. In this scenario, this review will contribute to the emergence of new and modern diagnostic and therapeutic methods to the healthcare of RA patients, and to our knowledge, this is the first review to evaluate data about myokines, osteokines, adipokines, and hepatokines on RA pathophysiology.

## 2. Materials and Methods

### 2.1. Focal Question

The focal question that was considered in our review was “What are the roles of adipokines, myokines, hepatokines, and osteokines on the pathophysiology of RA”?

### 2.2. Language

We included in our review only studies published in English.

### 2.3. Databases

**Figure 1:**
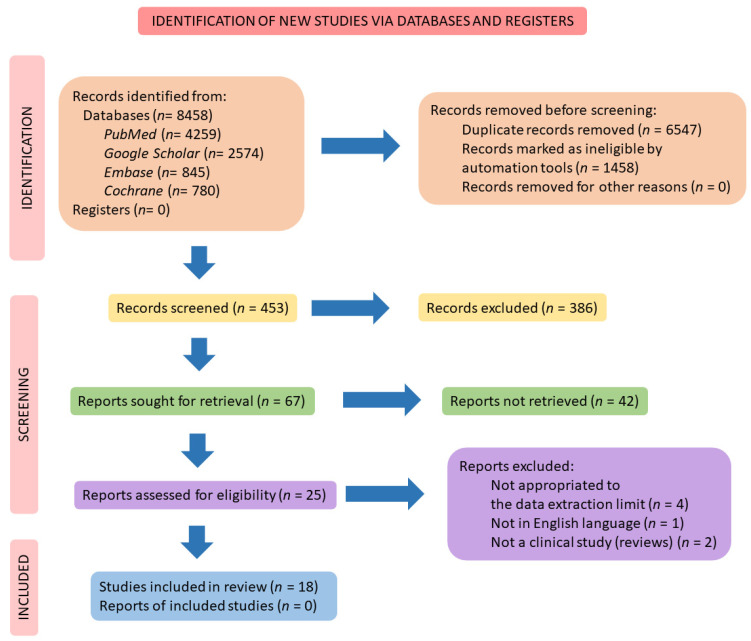
Flow diagram showing the literature search and study selection criteria. Analysis of the literature and writing of the manuscript were performed following the preferred reporting items for systematic reviews and meta-analyses (PRISMA) guidelines [12].

We consulted *PubMed, Embase, Google Scholar*, and *Cochrane* databases for building this review. The mesh terms used were “organokines” and “myokines” and “osteokines” and “hepatokine” and “adipokines” combined with “RA” and “arthritis” and “autoimmune diseases”. The mesh terms enabled the search and identification of clinical studies and other types of studies that were related to the objectives of this complete review. The preferred reporting items for a systematic review and meta-analysis (PRISMA guidelines) [12] were followed (Figure 1).

### 2.4. Study Selection

The inclusion criteria comprised case-control studies, observational cross-sectional studies, observational and longitudinal studies, case-control cohort studies, prospective cohort studies, Mendelian randomization studies, cohort analytical studies, post hoc analyses, large-scale cohort studies, and multicenter studies. Only full texts were considered.

The exclusion criteria were animal models, in vitro studies, studies published in a language other than English, reviews, poster presentations, case reports, and editorials. Descriptive and systematic reviews helped to build the discussion section.

### 2.5. Data Extraction

Only studies published from 2020 to 2022 were selected to compose this review. This data extraction was used due to the high number of studies evaluating organokines in RA. Therefore, the restricted time served as an updater for the issue.

## 3. Results

Figure 1 represents the scheme for the search for the included studies. From the 18 studies selected, more than 17,000 individuals presented the diagnosis of RA. The age range was over 18 years old. No clinical trials that did not include RA patients were selected for composing this review.

The studies included in this review are from Italy [13], Egypt [14,15,16], Unites States [17,18], Mexico [19,20,21,22], Sweden [23,24], China [25,26,27], Turkey [28], and Finland [29]. One study was multicentered [14].

The included clinical studies were conducted to evaluate the roles of several different organokines in RA. These organokines were adiponectin [18,23,29,30], chemerin [14,19,20,29], leptin [18,23,25,29,31], resistin [23,29,32], visfatin [23,29], vaspin [15], omentin [15], fibroblast growth factor 21 [17,18], osteoprotegerin [16], osteocalcin [16], fibrinogen-like protein 1 [17], sex hormone-binding globulin [18], myostatin [19], and irisin [15,16,22].

## 4. General Aspects of RA and the Role of Myokines, Osteokines, Adipokines, and Hepatokines

### 4.1. Pathophysiological Aspects of RA

Although the pathophysiology of RA is still not fully elucidated, many hypotheses have been postulated in recent years. The pathogenesis of RA initializes many years before the first symptoms of this disease occur, which are derived mainly from joint inflammation. Many risk factors are involved in the development of RA in a healthy patient. These are genetic and environmental although genetic factors contribute to 60% of the individual’s risk. As commented before, HLA summarizes the genetic risk for RA. Besides that, non-HLA alleles are also important, such as the gene responsible for producing protein tyrosinase phosphatases N22. Environmentally, smoking exerts in a dose-dependent association a major susceptibility for RA, especially when the individual at risk also possesses a genetic predisposition. Over smoking, many infectious agents and infections, menopause and pregnancy hormones, and poor diet also influence the emergence of RA in an individual, precisely [20,21]. Over these years, epigenetic modifications on the genome and environmental factors have led to modified self-antigens, the first objects of RA joint disorders. In RA, hyperplasia of the synovial cells and synovial infections can also trigger the release of pro-inflammatory cytokines that also modifies more self-antigens in joint inflammation [5,22].

As in other rheumatoid diseases, in RA, genetic susceptibility plays a vital role in developing the disease. Susceptible human leukocyte antigens DR1 and DR4 (HLA-DR1 and HLD-DR4, respectively) genes drive the immune system to recognize important self-proteins and other self-structures as non-self, which activates immunity to form a complex response against critical joint molecules. These actions of autoimmunity are mediated by antigen-presenting cells (APCs), such as dendritic cells, which are responsible for presenting the antigens to CD4+ lymphocytes. In this case, B lymphocytes are also activated sequentially to T-lymphocyte signaling, which is called co-stimulation [5,23].

Autoantibodies are highly involved in RA pathophysiology and its development. These are the rheumatoid factor (RF) and the anti-citrullinated protein antibodies (ACPA), which are frequently encountered in blood samples of RA individuals. However, when related to target self-antigens, many more auto-antibodies have been classified among patients with RA. High-affinity FR autoantibodies were related to inflammation and antigen trapping in synovial fluid and joints besides higher unfavorable prognosis in significant titers. RF responses use a large spectrum of immunoglobulins (Ig), such as IgA, IgG, and IgM. Just like the RF, ACPA auto-antibodies responses can also use these Igs. RF and ACPA are the most studied molecules studied in RA patients. However, ACPA seems to be more specific than RF in the pathogenesis of the disease due to the ability of citrullinated auto-antibodies to target citrullinated proteins and form with these proteins the structures called immune complexes, which are accumulated in the synovial fluid [7,33,34].

Innate immune cells are also massively encountered in the synovium of RA patients. These cells are the main ones responsible for persisting the articular inflammation of the disease. Besides that there are proliferating synovial fibroblasts in RA, the inflammatory process also causes synovium hypertrophy, leading to the formation of an abnormal joint tissue called pannus, which not only invades but also destroys the articular structures locally by the production of pro-inflammatory chemokines and cytokines as well as of matrix MMPs [2,24].

In RA’s early stages and progression, there are neutrophils, monocytes and macrophages, dendritic cells, natural killer cells, and innate lymphoid cells. Neutrophils produce reactive oxygen species (ROS), release pro-inflammatory cytokines and chemokines, and express enzymes that lead to citrullinated proteins. Monocytes and macrophages secrete pro-inflammatory cytokines and express MMPs that destroy joints and induce apoptosis of many articular structure’s cells, such as parenchymal and stromal. Natural killer cells are capable of producing granzymes that may contribute to the generation of new autoantigens, in turn causing direct damage to articular cartilage and contributing to bone destruction due to the stimulation that these cells exert on pro-inflammatory cytokines production by macrophages [2,25].

Recent research identified gut microbiota dysbiosis, increased exposure to air pollution, and reduced exposure to ultraviolet B radiation (UVB) as complementary risk factors for the development of RA. Dysbiosis of the densely colonized population of bacteria that inhabits human intestines can increase the activity of APCs by stimulating certain types of receptors, which are toll-like receptors (TLR), responsible for mediating the recognition of microbe patterns or nod-like receptors. Additionally, gut microbiome dysbiosis stimulates T-cell differentiation, amplificated mucosal inflammation, intestinal barrier permeability, and molecular mimicry. All these events promote the poor relationship between gut dysbiosis and RA, leading to higher APCs transformation. Reduced contact with UVB can trigger reduced vitamin D production that amplifies immunomodulatory actions. Dysregulation of this mechanism can lead to the emergence of RA in predisposed individuals. Finally, higher exposure to air pollution can trigger RA based on ROS production, which activates nuclear factor-kappa b (NF-kB). NF-kB can also stimulate T helper 1 cells to produce large amounts of inflammatory cytokines, such as tumor necrosis factor alfa (TNF-α), interleukin 1 (IL-1), and IL-6. The inflammatory mediators stimulate the maturation of macrophages in APCs. When joint macrophages are transformed into APCs, these cells have major opportunities to break up autoimmunity control and present self-antigens of joints to T cells, which migrate to articular structures and cause inflammation, leading to RA [5,26,27,31,32].

Pro-inflammatory cytokines are highly present in the serum of RA patients and principally in the interior of the joints. Inflammatory cascades in RA lead to the massive production of TNF-α, IL-1β, IL-1, IL-6, IL-12, IL-15, IL-18, IL-17, IL-23, and transforming growth factor-beta (TGF-β) as pro-inflammatory cytokines. These are crucial to up-regulate the autoimmune events in RA and to cause synovitis and the inflammatory destruction of the articular structures, which leads to incapacitating pain. Among other inflammatory mediators, there is a production of all families of chemokines in RA patients, including CXC, CC, CX_3_C, and C. Along with inflammation, the cellular antioxidant machinery reduces by reduced levels of nuclear factor-erythroid 2-related factor-2 (Nrf2). This factor helps to encode phase II antioxidant enzymes due to its capacity for binding effectively on antioxidant response elements. Nrf2 is also responsible for responding against inflammation and protects tissue against the harmful effects of inflammatory stimuli. Nowadays, RA is successfully treated with drugs that neutralize pro-inflammatory cytokines, such as TNF-α [30,35].

**Figure 2:**
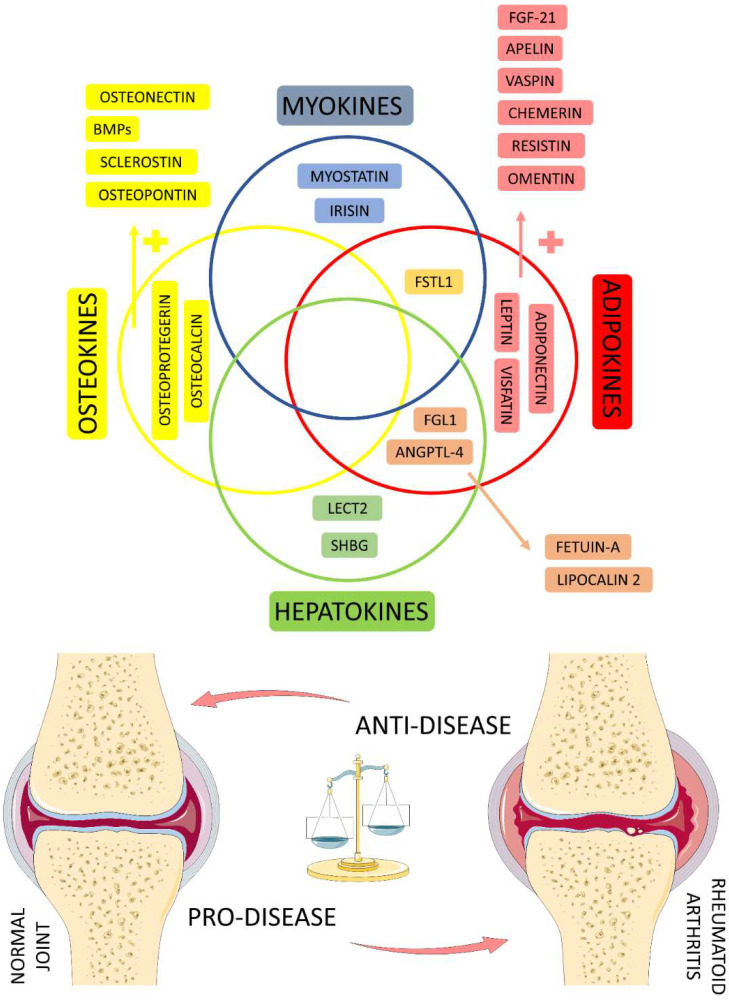
Representative scheme showing the main organokines and their balance in controlling the health and progression of RA disease. In general, organokines interact with different cells from different tissues involved in the pathophysiology of RA and, through these reactions, can lead these cells to develop inflammatory patterns, for example, or also produce and secrete metalloproteinases. BMPs, bone morphogenetic proteins; FGF-21, fibroblast growth factor 21; FSTL1, follistatin-like 1; FGL1, fibrinogen-like protein 1; ANGPTL-4, angiopoietin-like 4; LECT2, leukocyte cell-derived chemotaxin-2; SHBG, sex hormone-binding globulin.

In RA, synovial macrophages seem to be the most inflammation-related cells. These cells produce and release larger amounts of TNF-α, IL-1, and IL-6 pro-inflammatory mediators. The presence of these mediators stimulates fibroblast-like synoviocytes (FLS) and osteoclasts. Increased FLS cells relate to major productions of metalloproteinases, leading to cartilage degradation. In turn, increased maturation and activity of osteoclasts are related to augmented bone erosion. FLS also activates the NF-kB ligand (RANKL). RAKNL activated allows T cells to bind osteoclasts and increase bone erosion by stimulating osteoclast activity [5,33]. Figure 2 shows in a representative scheme the main organokines that will be discussed in this study and their balance in controlling the health or progression of the disease in RA patients.

### 4.2. Myokines in RA

Proteins secreted by skeletal muscles that play important roles in the body’s homeostasis are called myokines. Evidence suggests that these molecules can modulate endogenous liver gluconeogenesis, adipose tissue metabolism, thermogenic activities, pro-inflammatory stimuli, and insulin secretion from pancreatic β-cells. Myokines can administrate the proper metabolism of the skeletal muscles, adapting these organs to modification in energy needs, such as inactivity and sedentarism, and exercise. Although many authors diverge in the concept of which cells in muscles secrete mostly the high amounts of myokines, the myocytes appear to cover the secretion of a wider pool of these mediators. However, it is believed that muscle macrophages and other infiltrating immune cells can also be sources of cytokines secreted from muscles [36,37].

#### 4.2.1. Myostatin

Myostatin (growth differentiation factor-8, GDF-8) is the only known skeletal muscle cell inhibitor, playing critical roles in skeletal muscle cell proliferation and differentiation and in muscle physiology, controlling muscle fiber type transformation and protein synthesis and degradation. After humans are born, this myokine gene negatively regulates the growth and development of skeletal muscle fiber, limiting its number and size. More recently, being a member of the TGF-β superfamily, myostatin was related to metabolic pathways. This myokine affects fat and glucose metabolisms, affecting adipocyte proliferation and cardiomyocyte homeostasis. Additionally, myostatin also influences bone homeostasis and is associated with bone development [34,38].

Several studies have investigated the role of myostatin in RA pathophysiology. It was found that its levels are elevated in RA patients and that this myokine is associated highly with disease activity and inflammatory biomarkers presence, such as ESR. In synovial fibroblasts, myostatin upregulate TNF-α expression through the PI3K-Akt (phosphatidylinositol 3-kinase/protein kinase b) signaling pathway. Additionally, IL-1β expression is also augmented in synovial fibroblasts by myostatin through inhibition of the miR-21-5p signaling pathway. Remembering myostatin’s physiological roles and combining it to the elevated serum levels among RA individuals, myostatin in RA increases muscle atrophy and osteoclasts differentiation, promoting muscle degradation and bone destruction. This myokine can also stimulate Th-17 cells migration to the inflamed RA joints through the mechanism derived from the myostatin-CCL20-CCR6 axis [19,34,38,39,40,41,42,43].

#### 4.2.2. Irisin

Irisin is a skeletal myocyte-derived molecule produced and secreted while exercising and acts as a linker between skeletal muscles and other tissues, such as the nervous. This myokine is produced by the cleavage of the FNDC5 protein, which is presented in the membrane-bound of myocytes. Irisin’s physiological activities are mainly anti-inflammatory. This myokine downgrades inflammation through the inhibition of several different inflammatory pathways. Additionally, irisin regulates bone homeostasis by balancing bone resorption and bone formation and maintains bone mineral density. Moreover, it helps modulate metabolic processes principally related to the central nervous system and has a role in cancer proliferation, invasion, and migration. Under specific exercising conditions, irisin increases the white adipocytes’ browning augmenting thermogenesis and controlling weight [44,45,46]. In RA pathophysiology, lower levels of irisin are encountered. These low levels are related to augmenting inflammation and OS in RA via modulation of specific immune-inflammatory, necroptotic molecular, and biochemical pathways, such as the high-mobility group protein box 1 (HMGB1)/monocyte chemoattractant protein 1 (MCP1)/chitotriosidase I–mediated necroptosis. Besides, irisin concentrations in RA correlate inversely with disease duration, morning stiffness duration, and RA disease scores. It was also found that lower irisin concentrations in RA correlate significantly with cardiovascular diseases (CVD) and subclinical atherosclerosis formation [15,16,22,47].

### 4.3. Osteokines in RA

Osteokines are secreted by bone cells that possess effects locally in bones and systemically in other tissues and organs. They have endocrine, autocrine, and paracrine actions. In bones, osteocytes and osteoblasts are responsible for the secretion of osteokines through stimulation of several events, such as the mechanical stress from physical exercise. Osteokines exert healthy effects in increasing post-contraction glucose uptake in muscles through an insulin-dependent mechanism, influencing muscle contractility and mitochondrial biogenesis in the interior of muscle cells, reversing the decline in muscle function through the aging process, controlling phosphate homeostasis, influencing free fatty acids oxidation to produce energy, and mediating the transport of glucose transporters-4 (GLUT-4) from the cytoplasm to cell membranes. Among unhealthy effects, some osteokines can lead to the destruction and suppression of ectopic calcification of bones in addition to dysregulations in energy and phosphate homeostasis [10,48,49,50].

#### 4.3.1. Osteoprotegerin

Osteoprotegerin (OPG) is a soluble glycoprotein, a tumor necrosis factor receptor superfamily member. OPG is secreted by several cell types, such as osteoblasts, peripheral blood lymphocytes, endothelial cells, and vascular smooth muscle cells, and presents an important role in metabolism, being inversely proportional to adiposity and obesity. Inhibition of bone resorption is its most evident effect [51,52,53,54].

Osteoblasts secrete RANKL, directly proportional to the inflammatory marker CRP and promoted by inflammatory cytokines. RANKL binds to its receptor, nuclear factor κB receptor activator (RANK) on osteoclasts and monocytic osteoclast precursor cells, resulting in a stimulation of osteoclastic bone loss. The RANK link with RANKL is the main factor of bone destruction in inflammatory arthritis [52,55,56,57,58].

OPG has a protective effect on bone destruction and negatively regulates osteoclastogenesis by preventing RANK-RANKL binding by associating with RANKL, inhibiting osteoclast differentiation and activation, and increasing osteoclast apoptosis. As RANKL and OPG are the ultimate effectors of osteoclastogenesis, the ratio of RANKL to OPG in the bone marrow microenvironment is a key determinant of the rate of osteoclastic bone resorption. The balance between RANKL and OPG determines the degree of proliferation and osteoclast activity and reflects local bone loss around joints caused by inflammation. A low OPG/RANKL ratio is present in patients with RA compared to healthy patients and is associated with increased radiographic damage and joint and bone destruction in RA. In this sense, the RANKL/OPG relationship plays an essential role in regulating bone homeostasis in RA, and changes in this relationship may be a protective mechanism against accelerated bone loss in RA [47,51,52,53,55,56,57,58,59,60,61,62,63,64,65,66,67,68,69].

Several previous studies have investigated the relationship between OPG level and RA, with conflicting results. Some showed that RA patients had a higher OPG level than healthy controls, suggesting that elevated OPG levels may be a factor associated with RA. However, other studies have reported a decrease in OPG levels in RA patients, and some have demonstrated no difference in OPG indices compared to healthy RA patients and controls. Low levels of OPG in synovial fluid showed faster disease progression towards joint and bone destruction. Age, stage of the disease, and several other factors influence the levels of OPG, justifying such discrepant results between studies. Furthermore, autoantibodies to osteoprotegerin are associated with increased bone resorption in RA, with OPG antibody-positive patients having longer disease duration and activity and higher levels of bone resorption markers [66,67,70,71,72].

Significantly, OPG concentrations have been associated with the presence and the severity of coronary artery disease and predict future cardiovascular events, presenting an atherosclerotic role along with the inflammation characteristic of RA. Therefore, OPG highlights the potential implication of this molecule in the increased risk of CVD observed in patients with inflammatory arthritis. High amounts of OPG can be found in the arterial wall. Such a finding may suggest that endothelial cells may be significant contributors to the circulating pool of OPG in patients with RA [66,69,70].

Furthermore, it is interesting to highlight the crosstalk between several pro-inflammatory cytokines and OPG. Some of them regulate the expression of RANKL and OPG, including TNF-α and interleukins IL-1 and IL-6, which can decrease serum levels of OPG. All these factors are present in patients with RA, leading to a high prevalence of osteoporosis. On the other hand, other studies claim that TNF-α could increase the level of OPG or reduce it and induce osteoclast differentiation, increasing RANK/RANKL expression and resulting in osteoporosis [66,70,73,74].

#### 4.3.2. Osteocalcin

Osteocalcin (OCN) is a bone-derived hormone that is synthesized and secreted by osteoblasts and then activated by osteoclasts during bone resorption. It is a key factor responsible for the mineralization of the extracellular matrix, and its serum levels increase dramatically during this process, being used clinically as a marker of osteoblastic bone formation [13,75,76,77,78].

Serum OCN can enter distant cells to regulate IR, glucose homeostasis, and brain function. Its uncarboxylated and subcarboxylated forms (ucOCN) increase insulin sensitivity and secretion by directly stimulating the pancreas. It promotes the uptake of glucose and free fatty acids (FFA) by skeletal muscle and stimulates catabolism in skeletal muscle, increasing after physical activity and decreasing with aging. OCN levels are inversely associated with BMI, IR, C-reactive protein (CRP), and body fat mass. Its absence makes the brain smaller and less developed, particularly the hippocampus, and generates anxiety and compromises memory. At the same time, its presence would be effective against age-related cognitive decline and valuable in the acute stress response by inhibiting the parasympathetic system. Thus, serum OCN can enter distant cells to regulate IR, glucose homeostasis, and brain function, with anti-inflammatory and beneficial activity. On the other hand, some studies also claim that OCN can recruit osteoclasts and potentiate their chemotaxis and inhibit osteoblast activity, contributing to osteoclastogenesis and leading to osteoporosis [13,76,77,78,79,80].

During high disease activity in RA, specifically in acute RA, there is a decrease in bone formation markers, including osteocalcin. Its levels tend to be lower compared to healthy volunteers. Therefore, lower levels of this osteokine in acute phase RA patients can associate highly with bone destruction and articular changes [57,68].

#### 4.3.3. Osteopontin

Osteopontin (OPN) participates as a member of the small integrin-binding ligand (SIBLING) family of cellular matrix proteins. It was first identified in the bone matrix but was later discovered in almost all tissues. This organokine is an extracellular matrix glycoprotein present in the extracellular fluid surrounding the sites of mineralized tissue and bone remodeling. It is a substance strongly expressed in bone and released into body fluids, mainly secreted by osteoblasts. It is an important component of some events, playing an essential role in inflammation and bone metabolism. It acts as a pro-inflammatory mediator stimulating proliferation, migration, and adhesion of several cells and being an autocrine and paracrine factor. Its phosphorylated form is known to increase the number of macrophages and osteoclasts. In bone metabolism, its role is to promote the adhesion of osteoclasts to the mineralized matrix, regulating bone resorption and formation, being a substantial factor for cell adhesion and migration, and acting in the neuroendocrine regulation of bone mass. The absence of OPN can block OCN expression and the induction of mineralization. With aging, the expression of OPN in the bone marrow stroma is reduced. OPN is closely related to the development of many bone-related diseases, including RA [57,70,73,75,76,81].

Serum and synovial fluid OPN was significantly higher in RA patients compared to healthy patients and is correlated with markers of bone resorption in RA patients, and their high levels are directly proportional to serum levels of CRP, chemo-monocyte attractant 1 (MCP-1), macrophage inflammatory protein-1 beta (MIP-1β) on monocytes, interleukin-17 (IL-17), and other inflammatory cytokines including TNF-α and IL-1, IL-6, and IL -8. By binding to T cells, in addition to promoting the differentiation of Th1-type cells and increasing cellular immunity, OPN can, at the same time, inhibit Th2-type cells and humoral immune function. The imbalance of Th1/Th2 cells and the levels of secreted cytokines trigger events that lead to chronic inflammation and cartilage and bone destruction. Thus, OPN plays a crucial role in the pathogenesis and progression of RA by affecting the balance of Th1/Th2 cells and also inducing the differentiation of Th17 lymphocytes, affecting IL-17 levels and generating an inflammatory response, with its effects being more pronounced in their phosphorylated form [57,62,70,73,75,76,81,82].

OPN promotes osteoclast-mediated bone resorption by binding to its receptor, αvβ3 integrin, during arthritis. The binding of OPN to these cell surface receptors stimulates cell adhesion, cell migration, and other specific cell signaling functions, where OPN binds to fibronectin to activate FLS with B cells, stimulating the latter to produce inflammatory cytokines. It was also previously reported that it modulates tissue fibrosis by promoting TGF-β activation and fibronectin expression. Therefore, OPN acts as an important mediator in the maintenance of RA, as it activates synovial macrophages and fibroblasts, which stimulate cartilage and bone matrix degradation by secreting matrix MPPs and pro-inflammatory cytokines such as IL-6 and TNF-α in addition to stimulating fibrosis [73,75,76,81,82,83].

**Figure 3:**
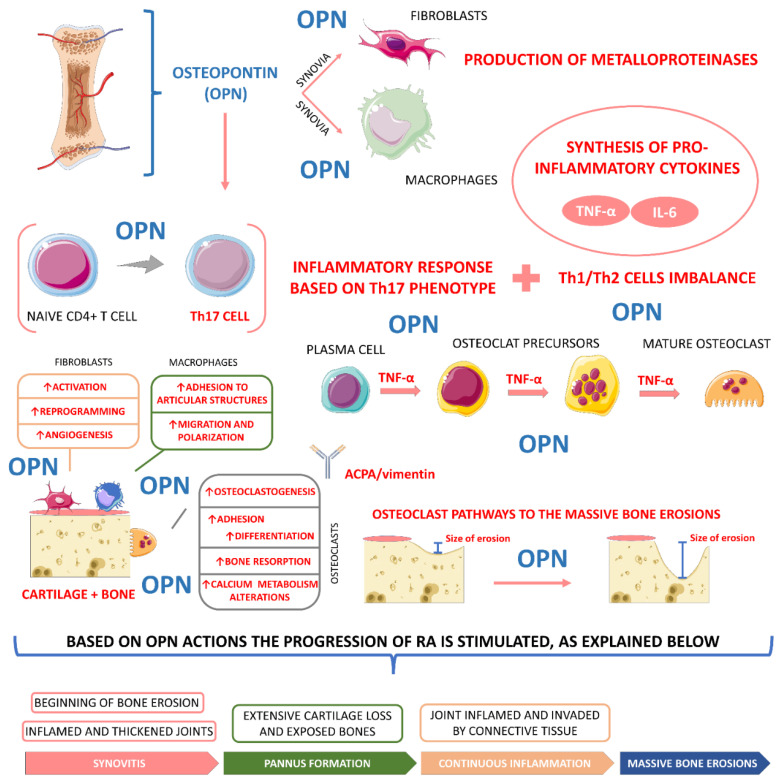
In bones, osteopontin is produced by specific induction, and in the synovium of joints affected by RA, it causes inflammation, production of metalloproteinases, and immune dysregulation. Mainly affecting macrophages, osteoclasts, lymphocytes, and fibroblasts, osteopontin is essential for the disease progression and the formation of bone erosions. ↑, increase; ↓, decrease; TNF-α, tumor necrosis factor alfa; IL-6, interleukin 6; Th17, T helper 17; CD4, cluster of differentiation 4; Th1, T helper 1; Th2, T helper 2.

Figure 3 presents the most critical actions of osteopontin in RA pathophysiology.

#### 4.3.4. Sclerostin

Sclerostin, the product of the SOST gene, is a secreted glycoprotein expressed primarily in osteocytes and chondrocytes and acts as a negative regulator of bone homeostasis by inhibiting bone formation by osteoblasts and stimulating osteoclast formation. It is an organokine that affects the activity of bone morphogenetic proteins (BMPs) and is an inhibitor of the Wnt/β-catenin metabolic pathway in bone cells. Although sclerostin is as an osteocyte-specific protein, recent studies have shown that several additional cell types express SOST and can produce the sclerostin protein, such as osteoclasts, chondrocytes, and in tissues such as the kidney, heart, and liver [73,84,85,86].

Sclerostin is a key protein that inhibits osteoblast activity and represents a crucial link between osteoblasts and the mechanosensory capacity of osteocytes, as the absence of charge leads to increased sclerostin expression and bone loss. Osteocytes produce sclerostin by modulating the TGF-β-dependent pathway in response to mechanical loads. Mechanical loads directly stimulate osteoblasts and mainly decrease the synthesis of cytoplasmic signaling molecules in osteocytes that are needed for the upregulation of SOST gene expression. Consequently, sclerostin production is reduced, which indirectly stimulates osteogenesis, with the loss of sclerostin generating a high bone mass phenotype [65,87].

TNF-α induces sclerostin expression in osteocytes and FLS in RA, which increases osteoclast formation. Furthermore, sclerostin blocked TNF-α-induced inflammatory activity, suggesting a protective role of sclerostin in chronic inflammation. Sclerostin plays an important role in osteocyte–osteoblast signaling. When incorporated into the bone matrix, osteoblasts transform into osteocytes and increase sclerostin expression. Cytoplasmic extensions then transfer it to osteoblasts located on the surface of bone trabeculae. Furthermore, as sclerostin increases RANKL mRNA expression and reduces OPG, activation of NF-κB occurs, which further activates genes necessary for osteoclast differentiation. In healthy bone, osteocytes balance osteolysis and osteogenesis by controlling sclerostin secretion. Thus, as previously described, intracellular signal transduction regulates the development, proliferation, differentiation, migration, and apoptosis of osteoblasts. SOST inhibits the Wnt signaling pathway. This pathway is an essential stimulus for osteoblastogenesis, matrix mineralization, and OPG levels and inhibits apoptosis and osteoclastogenesis, which are essential in cartilage and bone homeostasis. Inhibiting the Wnt pathway by SOST involves activating caspases and pro-inflammatory cytokines produced by the synovial membrane, increasing bone resorption, and stimulating soluble antagonists of the canonical Wnt/βcatenin signaling pathway with subsequent inhibition of proliferation, maturation, and differentiation of osteoblast progenitors. Sclerostin can also antagonize BMP signaling directly by inhibiting BMP-7 secretion, leading to intracellular retention and proteasomal degradation of BMP-7, and blocking BMP signaling selectively in osteocytes that simultaneously synthesize sclerostin and BMP-7 proteins [73,75,84,85,86].

Some authors have found elevated serum levels of sclerostin in patients with RA, and these levels are even higher in those patients with RA and osteoporosis. Increased serum levels of SOST indicate poor prognosis and resistance to treatment in these patients. Therefore, RA is characterized by reduced Wnt signaling. Given the importance of Wnt signaling in maintaining cartilage homeostasis, understanding the role of sclerostin is of great interest. Bone loss, erosion, and systemic osteoporosis with an increased risk of fractures are seen mainly in RA. Therefore, sclerostin inhibition is a powerful tool for improving bone repair in inflammatory arthritis [73,75,84,85,86].

#### 4.3.5. Bone Morphogenetic Proteins

Bone morphogenetic proteins (BMPs) belong to the TGF-β superfamily and have a wide range of effects on different cell types. Such proteins are potent regulators of cell proliferation, differentiation, and apoptosis. BMP signaling plays a vital role in osteoblastic and joint differentiation and induces bone formation by some components [61,77,79,84,88].

A chondroprotective role for different BMPs has long been proposed based on different in vitro experiments. Recently, greater attention has been given to BMP-7, also named osteogenic protein-1 (OP1), as a chondroprotective factor, which can be used as a medication for fractures. Its levels decrease with aging and are TNF-α-induced in addition to being increased in RA patients compared to healthy controls. BMP-2 indirectly decreases bone resorption by inhibiting the expression of IL-34, an inflammatory and osteoclastogenesis-stimulating cytokine, in synovial fibroblasts, thus contributing to antagonizing inflammation and bone erosions in RA. Osteoblasts highly expressed BMP-3 at sites of bone erosion, and its expression is induced by TNF-α, inhibiting osteoblast formation and function. BMP-4 and BMP-5 expression is significantly decreased in synovial tissue of RA patients compared to healthy controls and may be partially responsible for reduced bone formation at sites of bone erosion [83,88,89,90,91].

#### 4.3.6. Osteonectin (Acidic and Cysteine-Rich Secreted Protein, SPARC)

Osteonectin, or acidic and cysteine-rich secreted protein (SPARC), is a multifaceted matricellular protein involved in normal and pathological tissue remodeling. SPARC is a multifunctional regulator of soft tissue cells and has diverse biological effects, including controlling the spread, proliferation, migration, and synthesis of soft tissue cell-matrix proteins and binding to collagens and directly regulating their assembly. SPARC is highly expressed in normal tissues in bones, teeth, eyes, and at sites of wound repair and tissue remodeling [62,71,92,93,94].

SPARC is critical in supporting bone remodeling and maintaining bone mass. Furthermore, it can facilitate extracellular matrix degradation by stimulating the synthesis of MMPs in the superficial zone of arthritic cartilage. At the same time, in the middle and deep zones, SPARC can regulate chondrocyte proliferation, promote matrix synthesis, and improve mineralization due to its ability to bind collagen and hydroxyapatite, exerting an anti-inflammatory response, inhibiting the NF-kβ pathway, and having increased levels after exercise. Therefore, since it is produced in both zones, SPARC has dual roles in the resorption and regeneration of arthritic cartilage. The abnormal synthesis and degradation of SPARC can be attributed to cartilage and bone destruction, and when expressed by endothelial cells in the synovium during vascular remodeling, it releases a series of bioactive peptides that could regulate angiogenesis and result in synovial hyperplasia. SPARC decrease is related to increased inflammation and secretion of cytokines such as TNF-α due to intense activation of the NF-kβ pathway [71,92,93,94].

In RA, SPARC was increased in joint synovial cells, and the mean levels of SPARC in synovial fluid from RA patients were significantly elevated, being down-regulated by inflammatory cytokines. SPARC has been shown to exist in numerous chondrocytes in the superficial and middle zones of the cartilages of RA patients, whereas it is not found in these zones of normal cartilages [79,95,96].

### 4.4. Adipokines in RA

The adipose tissue (AT) is involved in the endocrine regulation of the body’s homeostasis and not only in the energetic homeostasis. Adipocytes drive the production and secretion of endocrine molecules (adipokines), which can control lipid metabolism and insulin sensitivity, inflammation, fibrogenesis, immunological responses, liver fat deposition, and fibrogenesis. Adipokines were the first organokines related to the pathophysiology of RA, collaborating in the inflammatory response on the affected joints. Adipokines also play a relevant role in developing extra-articular inflammation-dependent manifestations of RA. In musculoskeletal disorders such as RA, adipokines were described as modulators of bones, synovial membranes, and cartilages activities. Additionally, higher levels of adipokines are encountered in both serum and synovial fluid in patients with RA than in those who are healthy [6,7,72,74].

#### 4.4.1. Adiponectin

Adiponectin is composed of 244 amino acids produced and secreted by adipocytes to produce effects, mainly in the liver and skeletal muscle cells. Two adipokine receptors were found to respond to adiponectin, that is, adipoR1 and adipoR2. In health and against CVD, adiponectin exerts anti-inflammatory actions in obesity, atherosclerosis, type 2 diabetes mellitus, and metabolic syndrome, principally when in high concentrations. In muscles, the main effects of adiponectin are the increase in free fatty acid oxidation and glucose uptake. In the liver, adiponectin reduces gluconeogenesis. Paradoxically, in RA pathogenesis, the roles of this adipokine seem to be different [78,80,97].

Adiponectin in RA is pro-inflammatory to the joints, principally due to its ability to stimulate the production and secretion of inflammatory mediators. In RA patients, plasma and synovial adiponectin levels correlate positively with radiographic damage. Increased adiponectin concentrations promote inflammation by the production of TNF-α, IL-6, and IL-8. Interestingly, the erythrocyte sedimentation rate (ESR), CRP, and RF increase adiponectin concentrations in active disease RA patients. Many authors also suggest that baseline levels of adiponectin can predict the gravity of RA’s radiographic progression. In synovial fibroblasts, adiponectin induces the production of prostaglandin E2, MMPs 1 and 13, IL-6, and IL-8. In human chondrocytes, adiponectin seems to stimulate the production of nitric oxide, IL-6, IL-8, MMP-3, MMP-9, and the monocyte chemoattractant protein (MCP) 1. Adiponectin also shows effects on promoting differentiation of T cells from naïve to Th17 (T helper 17 cells) state, which contributes to synovial inflammation and increases bone erosion in RA patients, causing major deformations. Lymphocytes and endothelial cells respond locally in joints to the presence of adiponectin, causing inflammation. Synovial macrophages and synovial fibroblasts also are stimulated by adiponectin in the lining and sub-lining layers of joints, leading to more inflammation and angiogenesis (fibroblasts start to produce the vascular endothelial growth factor—VEGF). In angiogenesis, adiponectin-derived production of VEGF by synovium fibroblasts leads to endothelial progenitor cell formation and migration [8,9,10,74,78,98].

**Figure 4:**
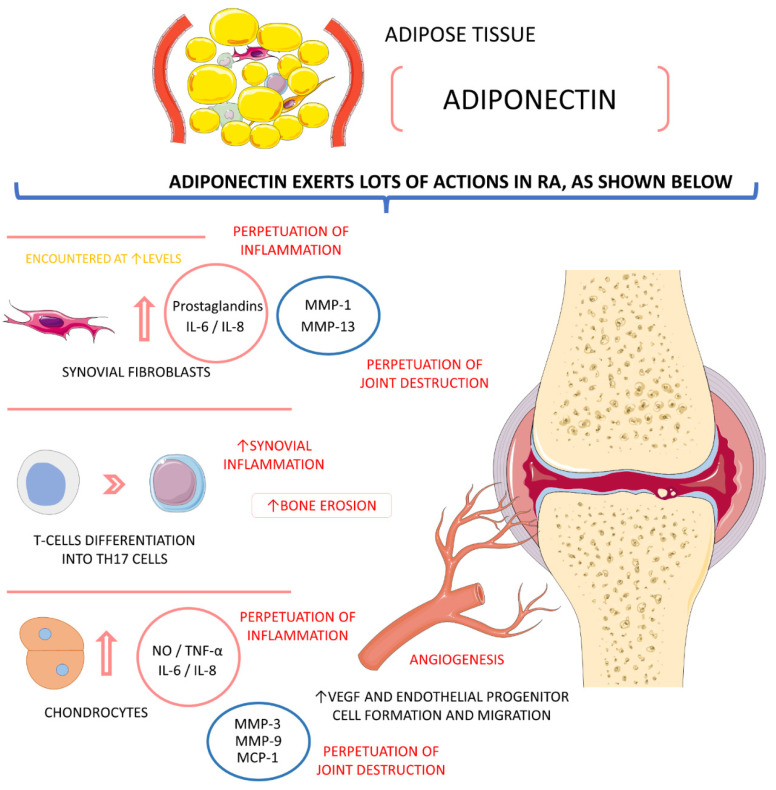
Adiponectin’s actions promote RA disease occurrence and progression. Although the major role of adiponectin in the body remains metabolically, adiponectin can also be associated with the development of rheumatological diseases. This adipokine promotes inflammation, immune dysregulation, and angiogenesis among RA patients, insofar as it can promote joint destruction. Particularly, angiogenesis can increase leukocyte influx to the interior of the joints. ↑, increase; ↓, decrease; IL-6, interleukin 6; IL-8, interleukin 8; MMP-1, metalloproteinase 1; MMP-13, metalloproteinase 13; Th17, T helper 17; NO, nitric oxide; TNF-α, tumor necrosis factor alfa; MMP-9, metalloproteinase 9; MCP-1, monocyte chemoattractant protein; VEGF, vascular endothelial growth factor.

Figure 4 shows the activities of adiponectin in RA pathophysiology.

#### 4.4.2. Leptin

Leptin is the main adipokine secreted by adipocytes and has a role in stimulating chronic, low-grade inflammation in obese individuals, increasing IL-6 and TNF-α production. In addition to its unhealthy inflammatory role, leptin is also related to decreasing the body’s sensitivity to adiponectin in obesity. Among other actions, this adipokine is implicated in regulations of basal metabolism, insulin secretion, reproduction, and bone mass. Its production is controlled by food intake, sex hormones, energy status, and inflammatory mediators. Leptin can also modulate both the innate and adaptive immune systems by activating proliferation and activation of macrophages and monocytes, regulating the cytotoxicity of natural killer cells, modulating neutrophils chemotaxis, and controlling T CD4, Th1 (T helper 1), and Th2 (T helper 2) cells’ phenotypes [11,99,100,101].

This adipokine was associated with obesity and CVD, increased disease course velocity, and increased disease activity and duration among RA patients. For these reasons, higher leptin levels were correlated with increased joint erosion. Leptin also modulates inflammation through JAK2/STAT3, NF-kB, and activating protein-1 (AP-1) pathways, maintaining positive correlations mainly with IL-17 in plasma and IL-6 and IL-8 in the synovial fluid of RA patients. Adhesion molecule production was also stimulated by leptin in human chondrocytes through JAK2, PI3K, and MAPK signaling pathways, which potentializes leukocyte invasion in inflamed joints [85,99,100,102,103].

#### 4.4.3. Visfatin (Pre-B-Cell Colony-Enhancing Factor—PBEF)

Visfatin/PBEF is an adipokine initially described as an early B-cell development cytokine and is secreted mainly by visceral adipocytes. Recently, visfatin/PBEF gained attention due to its relevant roles in neurological and oncological disorders a key inflammation regulator in these conditions. Visfatin/PBEF has also been related to the emergence of musculoskeletal diseases, such as RA. Although visfatin/PBEF can be secreted by other non-AT organs such as skeletal muscles, liver, lungs, kidneys, and bone marrow, its secretion is higher in adipocytes. In this review, it is considered only an adipokine. In RA patients, visfatin/PBEF can also be secreted by activated joint synovium, cartilage, and mononuclear cells, increasing joint inflammation. Visfatin/PBEF can also work as an enzyme. In summary, the main enzymatical roles of visfatin/PBEF depend on NAD+ vital cellular processes, which gives visfatin/PBEF nicotinamide phosphoribosyl transferase (NAMPT) effects. In metabolic diseases, visfatin/PBEF correlates with augmented IR and pancreatic β-cells dysfunction [10,104,105].

Pathologically, visfatin/PBEF in RA exerts many actions. This adipokine was related to up-regulating inflammation through signal transducers and activators of transcription 3 (STAT-3)-dependent IL-6 trans-signaling and poly(I-C)-mediated TLR-3 (TLR-3) pathways in activated RA synovium fibroblasts. In these fibroblasts, visfatin/PBEF activates the NF-kB, which leads to inflammation. Other inflammatory mediators are also produced through visfatin/PBEF stimulation in RA, such as IL-6, MMP-3, MMP-10, MMP-12, and MMP-19, which only augment an aggressive phenotype in RA synovium fibroblasts. Visfatin/PBEF also activates chondrocytes to produce prostaglandin E2 and MMP-3. These visfatin/PBEF effects on fibroblasts and chondrocytes up-regulate joint damage and destruction. In turn, visfatin/PBEF correlates intimately with RA disease activity and progression and its radiographic progression over four years of the disease. In RA, visfatin/PBEF also up-regulates the total number of circulating B cells, which can contribute to autoimmunity. In this field, CD14+ monocytes are stimulated by visfatin/PBEF to produce IL-6, TNF-α, and IL-1β. Many studies reported that visfatin/PBEF also mediates chemoattraction of immunological cells to the synovia by stimulating elevated expressions of adhesion molecules, such as vascular-cell adhesion molecule 1 (VCAM-1), intercellular adhesion molecule 1 (ICAM-1), and intercellular adhesion molecule 2 (ICAM-2), and chemokines, such as of CXC and CC clusters. VCAM-1, VCAM-2, and ICAM-2 promote attachment and migration of RA-activated synovium fibroblasts to cartilage via the CXC/CC enhanced cell motility. Attraction and extravasation of leukocytes are also promoted by visfatin/PBEF by stimulation of interstitial angiogenesis. This adipokine demonstrated pro-angiogenic effects by expressing extracellular signal-regulated kinase 1/2 (ERK 1/2) and fibroblast growth factor 2 (FGF-2) in endothelium-activated cells. The actions of visfatin/PBEF in RA are summarized in contributing to inflammation, matrix degradation, and angiogenesis [10,86,89,90,106].

#### 4.4.4. Omentin

Omentin is a glycoprotein adipokine first described in patients with bowel disease although it is found in the plasma of clinically healthy individuals. The visceral AT is the one that most secretes omentin. Besides other effects, omentin is described as cardioprotective and anti-atherogenic, mainly due to its vasculoprotective and vasodilatory actions. This adipokine is considered anti-inflammatory, modulating activation and proliferation of macrophages to the M2 phenotype. It is also negatively associated with metabolic syndrome. Low serum levels of omentin are associated with obesity, and high levels are associated with insulin sensitivity improvement. In RA patients, hypo-omentinemia is related to chronic inflammation. Added to that, low levels are encountered in the synovial fluid of RA participants. A positive and direct relationship was described between overweight and increased risk of RA development in individuals with positive autoantibodies for this disease. Omentin is also inversely correlated with MMP-3 production among RA individuals. A role of omentin against RA is that this adipokine can successfully decrease activation of Janus kinase 2/STAT3 (JAK-2/STAT3) pathways, which reduces inflammation and decreases MMP (metalloproteinase) production. To date, the roles that omentin exerts in rheumatic diseases are still unclear, and further researches are necessary to truly evaluate this adipokine in RA pathophysiology and progression [14,105,107,108,109].

#### 4.4.5. Resistin

Among obese patients, resistin is associated with the occurrence of IR and the development of CVD. The resistin actions can be related to its pro-inflammatory properties in obesity, mainly driving the production of TNF-α and IL-6 cytokines. In RA patients’ serum and synovial fluid, higher levels are associated with increased chemokine production by fibroblast-like synoviocytes, which contributes positively to the RA pathophysiology. Additionally, studies have demonstrated that resistin could have a pro-inflammatory role among RA patients, increasing mainly inflammatory biomarkers, such as CRP. Some studies also demonstrated that resistin can augment angiogenesis among endothelial progenitor cells due to VEGF increased production, which only facilitates RA pathophysiology in increasing possibilities to leukocytes’ migration into the articular synovial spaces of individuals with RA. Indeed, the association between resistin levels and leukocyte count, as well as IL-6 levels in the synovial fluid of RA patients, was found to be positive [10,74,91,95,96,110].

#### 4.4.6. Chemerin

Chemerin is a pro-inflammatory adipokine with endocrine, paracrine, and autocrine effects and is involved in the pathophysiology of many different metabolic disorders, such as metabolic syndrome, IR, and obesity. This adipokine is highly expressed in the white AT (WAT), liver, and lung tissues. However, chemerin can act as an anti-inflammatory under specific conditions. In contrast with other organokines that generally influence tissues, chemerin receptors are primarily expressed among immune cells [111,112].

In RA, chemerin induces FLS to produce metalloproteinases such as MMP-3, promoting cartilage damage and articular degradation. Chemerin is also associated with RA disease activity and severity, which helps predict disease progression. Among RA patients, chemerin promotes inflammation by inducing many pro-inflammatory cytokines, such as IL-6 and IL-1β. Besides MMP-3, other degradation-related molecules can be produced by chondrocytes stimulated by chemerin among RA patients, such as the C-C motif ligand 2 (CCL2). Combining inflammation and angiogenesis in RA pathophysiology, chemerin also stimulates motility and migration of immune and fibroblast cells to the joints, augmenting cartilage degradation [14,19,20,107,113,114].

#### 4.4.7. Vaspin

Visceral AT-derived serpin protease inhibitor (vaspin) is an adipokine that belongs to the serine protease inhibitors family. Although its secretion occurs mainly by the visceral and subcutaneous AT, vaspin is expressed among other organs, such as the liver, stomach, skin, and pancreas. Vaspin biological activities are related mainly to glucose metabolism, appetite control, and lipid profile control, protecting against diabetogenic gene expression and reducing local inflammation in AT. Vaspin also enhances insulin secretion and β-cells protection in the pancreas and promotes vascular function. In the blood vessels, it reduces pro-inflammatory stimuli and decreases the presence of vascular adhesion molecules. In turn, it promotes macrophage phenotype modification from M1 to M2 and decreases ROS production. The appetite is associated with a decrease in neuropeptide Y secretion and increases in energy expenditure. In the liver, it augments insulin half-life and promotes augmented insulin signaling [115,116,117].

In RA patients, vaspin seems to be elevated compared to controls. Due to this presence, this adipokine correlates extensively and positively with the inflammatory response of these individuals and is also associated with muscle inflammation among RA individuals. Along with other data, previous research conducted with RA symptomatic and non-symptomatic patients concluded that the serum levels of vaspin might be associated with RA symptomatology, reflecting disease activity and symptoms progression [14,107,118,119].

#### 4.4.8. Apelin

Apelin is an adipokine that intimately correlates with the cardiovascular system, helping control cardiac function (contractility) and blood pressure. Additionally, this molecule also plays an essential role in diabetes and obesity, being considered mainly in the progression of these two comorbidities. Produced principally by the AT, apelin was encountered in the brain, lungs, bloodstream, and kidneys. More recently, this adipokine correlated positively with RA pathophysiology [107,120,121].

Among RA individuals, apelin levels are decreased. This adipokine in RA and other rheumatic diseases increases the endothelial progenitor cell angiogenesis via inhibition of the miR-525-5p/angiopoietin-1 pathway. Additionally, apelin correlated positively with MMP-2 but inversely with MMP-9 among RA patients. In turn, in vitro studies using chondrocytes showed that apelin could promote the production of many metalloproteinase types, such as MMP-1, MMP-3, and MMP-9, added to inflammatory cytokines, such as IL-1β. However, the most important role of apelin in RA individuals seems to be related to the prediction of RA patients’ cardiovascular risk, insofar as apelin, along with the inflammation of RA, can be used to predict atherosclerosis development and atheroma plaque stability in RA patients.

FGF-21 is an organokine released mainly by AT and regulates glucose and lipid metabolism. In RA individuals, increased levels of FGF-21 are found, principally in seropositive patients. In many cases, FGF-21 can also stimulate bone resorption when in contact with muscles in response to insulin signaling. However, in RA, the roles of FGF-21 are mainly anti-inflammatory, decreasing macrophage mediate inflammation (suppressing Nrf2 and NF-kB) and pro-inflammatory cytokines secretion. FGF-21 decreases TNF-α, IL-1β, IL-6, IL-17, IL-2, MMP-3, and IFN-γ (interferon-gamma) levels and, on the other hand, increases IL-10. In RA, FGF-21 is also considered an ameliorator of the disease activity due to antioxidant and immunological actions. FGF-21 can inhibit oxidation due to disbalances between pro-oxidative/anti-oxidative enzymes. In turn, FGF-21 can decrease the activity of both cellular and humoral immune responses in individuals affected by RA. To maintain joint integrity, FGF-21 blocks the production of cathepsin K and metalloproteinases, especially the MMP-3 [11,122,123,124,125,126].

### 4.5. Hepatokines in RA

Hepatokines are hepatocyte-derived proteins that have been discovered recently as hormones. The secretion of these molecules is regulated by physiological or pathophysiological metabolic stressful conditions, such as fasting or exercise and obesity and IR, respectively. Improving or worsening metabolic conditions, hepatokines possess autocrine and paracrine actions besides endocrine, which means that these mediators alter the metabolism of the liver and other tissues and organs. [8,64,127,128]

#### 4.5.1. Leukocyte Cell-Derived Chemotaxin-2 (LECT2)

Leukocyte cell-derived chemotaxin-2 (LECT2) is an hepatokine that regulates immunological ad inflammatory responses in many events, such as sepsis, liver cancers, and hepatitis. Additionally called chrondomodulin-2, this molecule is very versatile, and it was first identified as a promoter of neutrophil migration to inflamed tissues. More recently, this hepatokine was correlated with regulatory actions on glucose metabolism, obesity, and the development of non-alcoholic fatty liver diseases. It also promotes fibrogenesis, vascular invasion, and tumor metastasis in cancer of various cell types [129,130,131].

LECT2 was associated with RA pathophysiology, involved in the dysregulation of osteoclasts, osteoblasts, chondrocytes, endothelial cells, and mesenchymal stem cells in the bone microenvironment. Although there is no correspondence in previous studies if LECT2 concentrations are elevated or not among RA patients, it is a consensus that LECT2 Val58Ile polymorphism is associated with elevated joint destruction. Other polymorphisms are responsible for the dysregulation of osteoclasts, osteoblasts, chondrocytes, endothelial cells, and mesenchymal stem cells in RA pathophysiology although these polymorphisms are still not known. The dysregulation of these cell joints is derived from the influx of pro-inflammatory immune cells to the articular spaces, increasing pro-inflammatory cytokines, such as IL-1β and IL-6, which are associated with bad joint prognostics in RA [104,105,130,131,132].

#### 4.5.2. Sex Hormone-Binding Globulin (SHBG)

There is a lack of studies regarding direct sex-hormone-binding globulin (SHBG) and its role in RA patients. SHBG is a glycoprotein that is primarily synthesized in the liver. Along with regulating free circulating levels of serum androgens and other sex hormones (e.g., estrogens), SHBG levels are associated inversely with the occurrence of diabetes and fatty liver diseases. In RA pathophysiology and progression, SHBG might have indirect positive effects at lower levels, mainly driving increases in androgens levels. It is sub-expressed, or its levels do not show significance compared to controls among RA individuals. In inflammatory affections, elevated free testosterone and dehydroepiandrosterone sulfates (DHEAs) levels may be related to anti-inflammatory actions. These levels of androgens can also promote immunoinhibitory actions in the inflamed RA joints, decreasing disease activity and progression [18,108,109,133,134].

Estrogens may exert pro or anti-inflammatory effects, stimulating immunological cells, such as B cells, or inhibiting macrophages and some types of T cells. When estrogens enter the equation, the situation changes due to the antagonistic actions of these feminine-sexual-related steroids hormones. In RA, elevated free estrogens levels due to reductions of SHBG can probably lead to RA progression. In rheumatic conditions, androgens–estrogens conversion in the inflamed tissues is increased, and the estrogens metabolites synthesis can support the disease occurrence. In contrast with estrogens, progesterone and progestogens also have similar effects in RA compared to androgens, as these molecules are intrinsic to the immunoinhibitory impacts [108,109].

**Figure 5:**
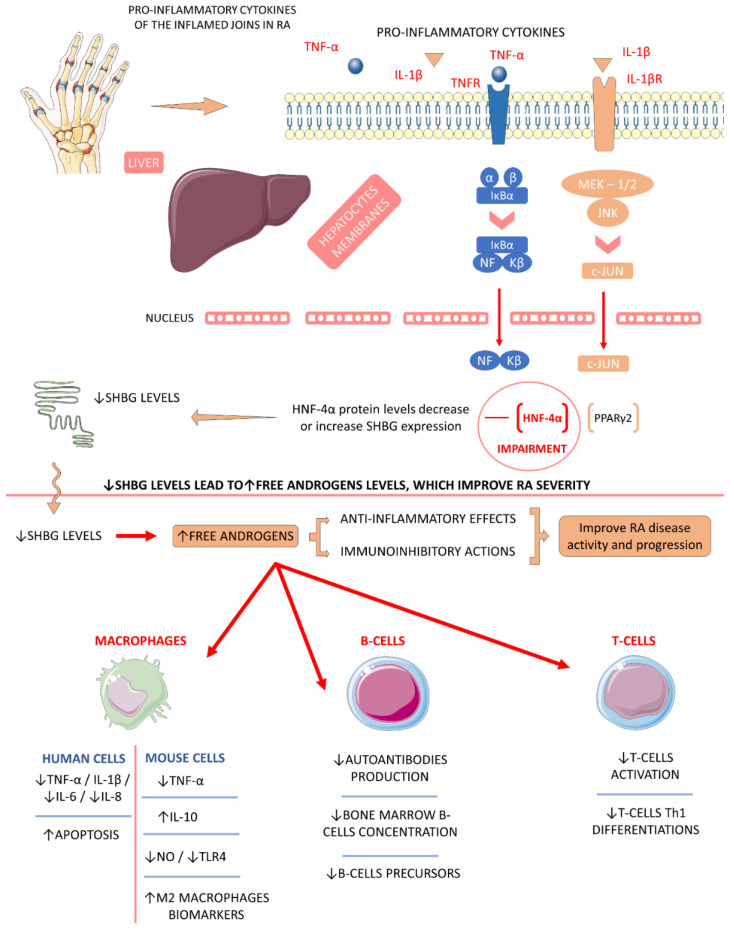
Particularly, the pro-inflammatory cytokines liberated by affected RA joints can lead the liver to decrease the production of its proteins, such as the sex-hormone-binding globulin (SHBG). While the levels of SHBG decrease, the levels of free androgens increase. These androgens can prospect anti-inflammatory and immunoinhibitory actions in RA pathophysiology, protecting the body against this disease and its harmful effects. The activities of the free androgens are mainly driven against macrophages’ inflammation and T-cell and B-cell dysregulation. ↑, increase; ↓, decrease; TNF-α, tumor necrosis factor alfa; IL-1β, interleukin 1 beta; TNF-αR, tumor necrosis factor alfa receptor; IL-1βR, interleukin 1 beta receptor; IkBα, nuclear factor of kappa light polypeptide gene enhancer in B-cells inhibitor alpha; MEK, mitogen activated protein kinase; JNK, N-terminal kinase; NF-kB, nuclear factor-kappa b; c-JUN, c-Jun N-terminal kinase; PPARγ2, Peroxisome proliferator-activated receptor gamma 2; HNF-4α, hepatocyte nuclear factor-4 alpha; IL-6, interleukin 6; IL-8, interleukin 8; IL-10, interleukin 10; NO, nitric oxide; TLR4, toll-like receptor 4; Th1, T helper 1.

Figure 5 shows how SHBG in lower levels can be associated with protection against RA disease occurrence and progression.

### 4.6. Organokines Released by Different Organs: Possible Crosstalk?

The adipose tissue, liver, bones, and skeletal muscles are the main organs that produce respectively adipokines, hepatokines, osteokines, and myokines. These biomarkers, which can be harmful or beneficial for RA pathophysiology, can still perform crosstalk and act together in the RA’s development. The gathering of knowledge about these molecules and their crosstalk is essential to evaluate whether organ interactions may drive autoimmunological or rheumatic conditions [7,135].

#### 4.6.1. Fibrinogen-like Protein 1 (FGL1)

FGL1 is a liver and AT-derived protein that is mitogenic for hepatocytes and promotes adipogenesis. This hepatokine/adipokine leads to hepatocyte proliferation via a specific extracellular signal-regulated kinase, the extracellular signal-regulated kinase1/2 (ERK1/2), which depends on autocrine mechanisms. In patients diagnosed with type 2 diabetes mellitus, FGL1 levels are higher than in healthy subjects and have a role in IR. Additionally, it is known that FGL1 has a role in developing non-alcoholic fatty liver disease although studies highlight that FGL1 has liver-protective effects against injuries. In recent years, FGL1 started to be considered an adipokine, suggesting crosstalk between liver and AT through this protein. In AT, FGL1 promotes mitogenic and lipogenic activities on adipocytes. It also increases preadipocyte proliferation and, in this case, facilitates lipogenesis. In 3T3-L1 adipocytes, FGL1 induces adipogenesis through an extracellular signal-regulated kinase 1/2—CCAAT/enhancer-binding protein β (ERK1/2-C/EBPβ). FGL1 also has a role in obesity and is considered a novel therapeutic target to combat this condition due to FGL1 effects on individuals at an elevated risk of being obese [136,137,138].

In RA patients, FGL1 exerts a role in positively predicting the activity of the disease and its prognosis in different patients. Due to these attributions, FGL1 can be useful in the daily clinical practice of predicting RA progression. Importantly, FGL1 also has immunomodulatory actions and is associated as an immune checkpoint target. In RA pathophysiology, FGL1 has been an important inhibitory ligand for the lymphocyte-activation gene 3 (LAG-3), an inhibitory immune receptor in antigen-specific T cells. The association of upregulated FGL1 in combination with assessment of disease activity is benefited by inhibition of autoimmunity. Additionally, LAG-3+ regulatory T cells were in lower levels in the serum of RA patients that presented high disease activity than in healthy subjects. Due to these attributions, FGL1 can be used as a possible intervention target in treating RA with different disease activities [26,139,140,141].

#### 4.6.2. Angiopoietin-like 4 (ANGPTL-4)

Angiopoietin-like protein 4 (ANGPTL-4) is a multifunctional protein with signaling attributions that is produced by a great variety of tissues, such as AT and the liver. It is mainly responsible for stimulating angiogenesis and performing metabolic crosstalk between many organs like the liver, AT, skeletal muscles, and brain. ANGPTL4 can control the increase in fatty acid oxidation and regulate the lipoprotein lipase activity, decreasing mainly the triglyceride metabolism and clearance, which leads to inflammation. Among diabetic individuals, this organokine regulates glucose levels and improves glucose tolerance, helping diminish, in some ways, the cardiovascular risk [142,143].

In RA patients, ANGPTL-4 influences bone resorption, cartilage destruction, and angiogenesis and has controversial roles in the RA inflammatory response. ANGPTL-4 is overexpressed in RA, principally in the synovial fluid, cartilage, and bones. In the cartilage and under inflammation-induced hypoxia, ANGPTL-4 stimulates the production of metalloproteinases, such as MMP-3 and MMP-1, by chondrocytes, which leads to the cartilage matrix degradation through catabolism processes. In the bones, inflammation-derived hypoxia stimulates osteoclasts to bone resorptions, principally in RA serum elevated RANKL levels. These actions of ANGPTL-4 on bones and cartilages derive from the combination of this organokine with the hypoxia-inducible factor (HIF). This factor induces ANGPTL-4 production and secretion by the cells involved in bone resorption and cartilage degradation. In RA, it can promote angiogenesis through inhibition of endothelial cells apoptosis and stimulation of endothelial cell migration; however, there is a disagreement among researchers if ANGPTL-4 promotes or inhibits angiogenesis in RA patients. In the inflammatory field, there are also controversies regarding whether ANGPTL-4 enables pro or anti-inflammatory effects in RA pathophysiology. Under the inflammation of RA, ANGPTL-4 is overexpressed, but there is no evidence that this organokine stimulates or not the production of inflammatory cytokines or chemokines. What is known is that ANGPTL-4 correlates positively with CRP levels in inflammatory conditions, such as metabolic syndrome and chronic pulmonary obstructive disease. This scenario cannot be different in RA patients [113,114,144,145,146].

#### 4.6.3. Fetuin-A

Fetuin-A is both an hepatokine and an adipokine although hepatocytes predominantly secrete it. This molecule is a multifunctional glycoprotein that imposes metabolic effects such as obesity, peripheral insulin sensitivity decrease, diabetes, and non-alcoholic fatty liver diseases, mainly at higher levels [11]. In autoimmune diseases such as AR and osteoarthritis, fetuin-A probably helps stimulate the production of pro-inflammatory cytokines by macrophages and other cells. Higher levels of this adipokine also correlate with inhibitory actions against adiponectin effects. Inflammation may also be driven by fetuin-A since it can activate TLR-4 and mimic the TGF-β receptor [118,119,147].

#### 4.6.4. Lipocalin 2

Lipocalin 2 (LCN2), also known as neutrophil gelatinase-associated lipocalin (NGAL), is a glycoprotein recently identified as an organokine derived from the AT and the hepatocytes. It can act as an inflammatory biomarker that positively correlates with BMI and metabolic syndromes. It was observed that LCN2 was expressed in several other cells, such as uterine cells, immune cells, spleen, kidney, and bone marrow. Different studies have reported its role in apoptosis, cell differentiation, fatty acid and iron transport, inflammation regulation, immune response, and metabolism [148,149].

In joint tissues, LCN2 expression is induced after stimulation of inflammatory factors and in response to mechanical loading. Once there is an increased expression of the LCN2 gene in AT of obese animals and a decreased expression of the LCN2 gene in obese animals receiving antidiabetic drugs, the idea that this protein is a pro-inflammatory agent is reinforced. The tissue distribution and expression of LCN2 in neutrophils, bone marrow, and tissues exposed to microorganisms, such as the trachea, lung, stomach, salivary gland, and colon, indicate its involvement in inflammatory responses. In neutrophils, LCN2 secretion is highly regulated by infection and activation of inflammation; lipopolysaccharide (LPS) and TNF-α are the two strong inducers of its production [148,149].

Serum levels of LCN2 were significantly higher in patients with RA than in healthy subjects and were similar between RA subgroups with high, moderate, and low disease activity. Significantly, these levels did not correlate with laboratory and clinical parameters of disease activity. In the same way, no correlation was found between joint structural damage and serum levels of LCN2. These results indicate that serum levels of LCN2 can be used to indicate structural damage, such as erosions in the early stage of the disease, but cannot be used to monitor disease activity and are not directly related to inflammatory activation. LCN2 is also believed to play a role in degenerative joint diseases and has been suggested as a biomarker of cartilage degradation in arthritic diseases [148,149].

#### 4.6.5. Follistatin-like 1 (FSTL1)

Follistatin-like 1 (FSTL1) is both an adipokine, myokine, and cardiokine. It is a glycoprotein characterized partially as a homologous of the follistatin family. This organokine is typically involved in many fields of human diseases, such as metabolic, cardiovascular, and rheumatic conditions. It is involved in inflammatory regulation and fatty acids and glucose oxidation among dysfunctional metabolic individuals. Additionally, FSTL1 is involved in cardiomyocytes hypertrophy and apoptosis and vascular smooth muscle cell proliferation and migration. It can also influence endothelial cells’ homeostasis, composing their survival, migration, and differentiation through the blood vessels of the whole body. More recently, FSTL1 was implicated in tumor growth and cancer metastasis [149,150,151].

In RA patients, FSTL1is usually encountered at higher levels compared to controls. These higher levels correlate intimately with elevated articular degradation and joint inflammation in RA and FLS proliferation, migration, invasion, and inflammation. In RA bones, FSTL1functions as an osteoclastogenic factor and osteogenic suppressor. It activates osteoclast proliferation via RANKL-mediated NF-κB activation and M-CSF-induced (macrophage colony-stimulating factor) precursor and suppresses osteoblast differentiation via inhibition of bone marrow mesenchymal cells. FSTL1 also up-regulates IFN-γ signaling pathways during RA progression and bridges both innate and adaptative immune responses. MMP-1, MMP-3, and MMP-13 gene expression are also up-regulated by FSTL1 among RA patients, principally by JAK/STAT3 and NF-κB signaling pathways [127,128,152,153,154,155].

**Figure 6:**
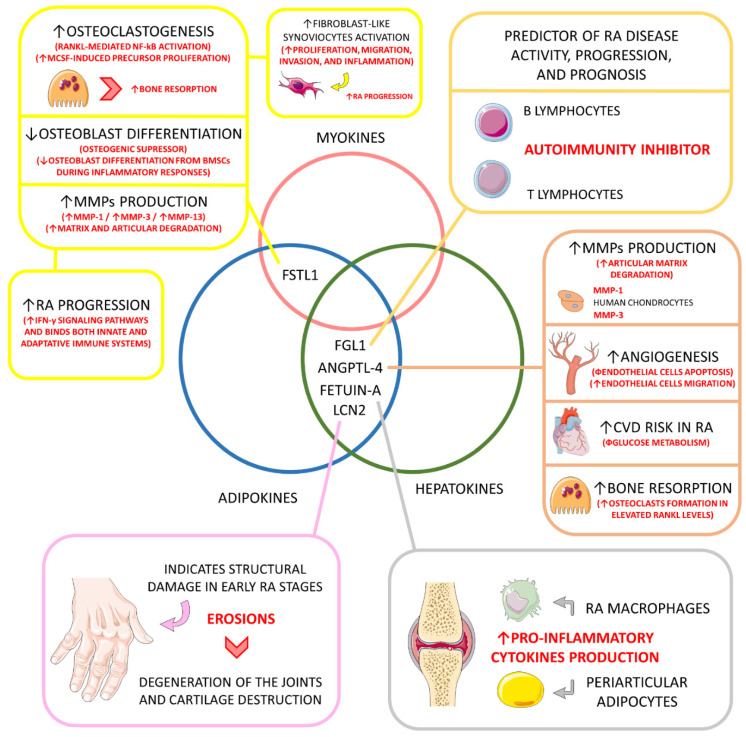
Organokines with simultaneous classifications that are involved in the pathophysiology and progression of RA disease. By acting together and combined, organokines can potentialize health or disease and lead to augmented disease activity. ↑, increase; ↓, decrease; Φ, inhibition; RA, rheumatoid arthritis; RANKL, receptor activator of nuclear factor kappa-Β ligand; NF-kB, nuclear factor kappa b; MCSF, macrophage colony-stimulating factor; MMP-1, metalloproteinase 1; MMP-3, metalloproteinase 3; MMP-13, metalloproteinase 13; IFN-γ, interferon gamma; MMP. Metalloproteinases; CVD, cardiovascular diseases; FSTL1, follistatin-like 1; FGL1, fibrinogen-like protein 1; ANGPTL-4, angiopoietin-like 4; LCN2, lipocalin 2.

Table 1 summarizes the main characteristics of organokines with simultaneous classification involved in the pathophysiology and development of RA and its progression. Figure 6 shows the organokines with simultaneous classifications involved in the pathophysiology and progression of RA.

### 4.7. Studies Evaluating the Role of Organokines in RA Patients

Some clinical trials showed the role of organokines in RA. Cheleschi et al. [13] conducted a case-control study with 50 RA, 50 psoriatic arthritis (PsA), and 50 controls to evaluate the roles of adiponectin, chemerin, leptin, resistin, and visfatin associated with microRNAs on discriminating RA from PsA. The results showed that leptin and a specific microRNA called microRNA 140 were increased more among PsA than in controls or RA patients. Additionally, TNF-α was higher in PsA patients than in only RA, which can also corroborate understanding of the diagnosis of these two rheumatic affections.

Wahba et al. [15] conducted a study to assess the role of the adipokines chemerin, apelin, vaspin, and omentin in RA pathophysiology and their genetic variants, named rs17173608, rs2235306, rs2236242, and rs2274907, respectively. Three hundred individuals participated in this study: 150 had RA, and 150 were healthy. The results showed that the adipokines chemerin and vaspin were at higher levels in RA patients than in healthy individuals and are associated with the clinical and laboratory parameters of this disease. Besides, apelin and omentin levels were lower in RA individuals than in healthy participants. Although this study presented results in four different adipokines, it has limitations. Firstly, the sample size of RA patients was drawn from established RA and in-treatment patients, affecting comparisons between adipokines and serum biomarkers of RA, such as CRP and the ESR.

Gould et al. [122] evaluated longitudinally the associations between FGF-21 and body composition changes in RA and the physical function of the 113 enrolled RA patients. The results showed that FGF-21 is associated with obesity and inflammation in the studied RA individuals, worsening physical functioning among RA. These findings are important in identifying rheumatic patients at risk of developing functional decline. However, this study has some limitations. Firstly, just 84 individuals (74% of the initial participants) completed the follow-ups, representing more than 20% of the sample’s loss. Due to this, larger studies are necessary to evaluate FGF-21 role in physical functioning, body compositions, and inflammation among RA patients.

Baker et al. [162] determined the associations between serum adipokines levels and abnormal body composition rates among 419 individuals diagnosed with RA. Adiponectin, leptin, and FGF-21 were the assessed adipokines. These molecules were associated positively with both excess adiposity and low rates of lean mass among RA patients. These results try to improve the prediction of body composition abnormalities and metabolic alterations in the pathophysiology of RA, which can be helpful in clinically predicting an individual’s risk of adverse outcomes, such as cardiovascular.

In an observational cross-sectional study, Vazquez-Villegas et al. [163] evaluated the chemerin serum levels and the occurrence of functional disabilities among women with RA. In all, data from 82 patients (35–75 years old) were analyzed, and 43 of them presented at a minimum of one functional affection. The final results concluded that higher chemerin levels were associated with more functional disabilities among these women, whereas no other blood biomarkers correlated with loss of function were present. However, some limitations are associated with this study. The serum levels of chemerin were determined transversely in the women; therefore, these authors do not have information about chemerin levels in patients who underwent treatment.

Gonzalez-Ponce et al. [164] amplificated the results of the anterior study by implicating chemerin levels as a biomarker of joint inflammation in RA women. In an observational cross-sectional study, these authors identified in 210 patients (56.59 ± 11.25 years old) that chemerin serum levels were highly and positively associated with RA inflammation and moderate and severe RA diseases.

Zhang et al. [165] conducted a case-control cohort study with 82 obese individuals with RA to identify if adiponectin and other adipokines could be associated with RA risk development. The results showed that adiponectin in higher levels was associated with an increased risk of RA development but not independently of other adipokines. In addition, elevated serum levels of leptin, resistin, and visfatin were not associated with elevated RA risk. Although this study innovates treating obese patients, its two cohorts (cases and controls) were not similar at baseline characteristics and proportionally.

Wahlin et al. [16], in a prospective cohort study, evaluated the possible interactions between subclinical atherosclerosis and markers of bone turnover, regulators of bone formation, and bone mineral density (BMD) in 79 patients on new-onset with RA (≤60 years old at the diagnosis). The subclinical atheroma formation was assessed by intima-media thickness (IMT), and the markers of bone formation and bone turnover were the osteokines osteopontin and osteoprotegerin. The results showed that osteoprotegerin and osteopontin were significantly associated with the subclinical atherosclerosis development in RA patients but not the BMD or other markers reflecting ongoing bone turnover.

Based on international large-scale genome association studies (GWAS), Hanzhu Chen et al. [14] were able to quantify the genetic correlation between adiponectin levels and RA. By bidirectional Mendelian randomization analysis, the authors found no evidence supporting a causal association between adiponectin and RA risk. The instrumental variables of this study (*n* = 67,739) were selected from a recent GWAS. RA patients were selected from a large-scale GWAS of 14,361 cases and 43,923 controls. Data of more than 11,437 cases and 604,953 controls were selected from databases to replicate the results and decrease bias risk,.

Chen et al. [166] conducted an analytical cohort clinical study with 223 RA individuals to assess whether serum leptin levels were more associated with cardiovascular events among RA patients. The results showed that compared with RA patients with leptin levels below the median value, RA patients with serum levels of leptin above the median value had higher cardiovascular affections prevalence with great significance.

Taylan et al. [28] also conducted an analytical cohort study with 47 RA and 25 control individuals to assess leptin serum levels associated with RA disease activity. The results demonstrated that the higher leptin serum levels were, the higher the disease activity also was. However, the authors concluded that treatment with disease-modifying anti-rheumatic drugs (DAMRDs) was associated with reductions in leptin serum levels.

Vuolteenaho et al. [29] conducted a post hoc analysis of an investigator-initiated, multicenter, controlled study. The authors realized whether resistin could be associated with not only inflammation but also disease activity among DMARDs-naïve RA patients as well as disease progression. In all, 99 RA and DMARDs-naïve patients were analyzed. The results showed that serum pretreatment resistin levels were significantly associated with a higher risk for RA patients of developing erosions in the early stages of the RA disease.

Liu et al. [17] conducted a large-scale cohort two-center study to evaluate if FGL1 can be used to predict RA disease activity and prognosis. This clinical study counted 1244 participants majorly diagnosed with RA. The results showed that FGL1 could positively predict RA disease activity and prognosis, a proper clinical predictor for RA progression. Although this trial had a large sample size, limitations existed. The recruited patients had at least six months of diagnosis, and it remained unclear if FGL1 plays a role in the early diagnosis of RA. Additionally, this study evaluated protein biomarkers alone, besides other metabolic biomarkers.

Qu et al. [18] conducted a Mendelian randomization study in 28,837 European ancestry individuals to evaluate whether the concentrations of SHBG were correlated with an elevated risk of RA development. The results showed a positive causal effect of circulating SHBG levels to predict RA with no sex specifications. This study also evaluated SHBG associations with osteoarthritis and ankylosing spondylitis.

Murillo-Saich et al. [19] conducted a cross-sectional clinical study with 84 RA women to evaluate the serum myostatin levels and RA inflammatory activity. The results showed that myostatin is significantly associated with inflammatory parameter concentrations and highly with RA disease activity. As this study was transversal, longitudinal alterations of myostatin levels among the RA women and the possible correlated implications were not assessed. Finally, the prevalence of rheumatoid cachexia among some patients limited the statistical power to compare myostatin levels and RA activity among same-diagnose patients.

Gamez-Nava et al. [139] conducted a cross-sectional study with 148 women with RA to evaluate the associations between serum irisin levels and osteoporotic vertebral fractures in these patients. The results were significant in pointing to lower irisin levels as a risk factor for the fractures. Although this trial was only conducted in women, it gives information about how lower levels of organokines can lead to incapacitant events, such as fractures, which are risk factors, such as falls.

Gamal et al. [167] conducted a preliminary cross-sectional study with 58 RA patients matched with 30 healthy controls to evaluate the association between serum irisin concentrations and the quality of sleep as well as to assess the disease activity of RA combined with irisin levels. The results showed that irisin levels were decreased among RA individuals and correlated inversely with the disease duration and morning stiffness in RA diseased. Added to this, lower irisin levels correlated significantly with poor sleep quality in RA patients. However, this is a small sample size to assess information about an organokine and sleep quality although other neurological causes for sleep disturbances were all excluded during this study screening.

In a cross-sectional study, Soliman et al. [156] evaluated if serum irisin concentrations correlate significantly with cardiovascular risk factors and subclinical atherosclerosis occurrence in 60 RA patients. The results showed that decreased irisin levels are significantly associated with cardiovascular deterioration and atheroma formation, showing irisin as a predictor of subclinical atherosclerosis. However, this study was cross-sectional, which decreases its generalizability to the detriment of longitudinal studies.

Table 2 shows the studies that evaluated the role of organokines in humans.

### 4.8. Future Perspectives about the Role of Organokines and RA

Genetic variations among different RA patients may interfere with the expression, production, and secretion of organokines. In this field, these gene polymorphisms can also interfere with the predisposition of RA to contact with other affections, such as CVD, which augments the risk of sudden death and reduce the quality of life. Leptin, as an example, is an adipokine that associates highly with polymorphisms and is inclusive in its receptors. Along with the world’s different populations, specific types of genetic alterations may be found, and these may be or not be related to the RA disease activity, such as in the Chinese population, in which the leptin genes polymorphisms rs10244329, rs2071045, and rs2167270 are all not associated with RA genetic susceptibility [140]. Organokines secretion can also be affected by a polymorphism in genes of apolipoproteins (APO). The specific APO E ε2ε3 genotype is associated with lower levels of TNF-α, IL-6, resistin, and visfatin among RA patients. RA patients with the APO E genotype ε3ε4 present higher LDL-c, IR, and CVD levels. The genotype ε2ε3 also correlates with decreased CVD risk among RA individuals [141,168].

Since RA is three times more common in women than men, it is possible that, in addition to the already known hormonal differences, there may also be a difference in the profile of organokine secretion in the different sexes affected by this autoimmune disease.

The secretion of organokines can also modulate systems such as the neural and the immune. These neuroendocrine and neuroimmunological axes influence and are influenced by the secretion of organokines. Some of them can be related with the intensity of pain present in both osteoarthritis and RA, and we believe that this is a great field to be explored.

In a chronic inflammatory disease such as RA, the interplay between bone formation and resorption is fundamental to bone homeostasis. Bone and joint integrity are regulated by a delicate balance of catabolic and anabolic immune and inflammatory mediators influencing the maturation and function of osteoblasts and osteoclasts. The organokines are certainly involved in this balance in different phases of the disease.

The concentration of some adipokines (adiponectin and visfatin/PBEF) correlates positively with radiographic damage. How many and/or which other organokines may also be associated with radiographic progression and thus incorporated in future clinical practice? We also know that severe obesity and exacerbated weight loss are associated with a rapid progression of disability in RA, including greater radiographic progression. Certainly, in obesity, there is an altered profile of organokines secretion, especially adipokines, which is most likely related to the development of the disease [169]. Obesity is also related to fat liver deposition. An unbalance in the synthesis and secretion of adipokines can also lead to metabolic syndrome and other liver diseases related to obesity and inflammation, such as non-alcoholic fatty liver disease (NAFLD) and non-alcoholic steatohepatitis (NASH). For these reasons, some questions cannot be ignored. It would be plausible to admit that in synovitis (joint inflammation), the local production of organokines could contribute to the pathophysiology of systemic manifestations, such as those that occur in the lung and other organs.

As for hepatokines, we know that some of them, such as sex-hormone-binding globulin, play an indirect role in developing RA. Thus, there is doubt about whether menopausal women (who have changes in the levels of this organokine) may have a different evolution or even a different prognosis for the disease. In addition, several studies have pointed out that these women often have increased visceral fat and MS. Therefore, we can propose that these patients produce organokines with pro-inflammatory activity, which could contribute to the progression of the disease.

We believe that the “good organokines” can inhibit the inflammatory process present in RA and contribute to the resolution of this inflammatory process and the well-known resolvins (lipid mediators derived from fatty acids, which act in the resolution of inflammatory processes). In this work, it was seen that organokines could interfere in the change of pattern from M1 (macrophage) to M2. A better understanding of how this change works is a challenge to be overcome to propose new solutions for the clinical management of the disease.

Moreover, possibly, the biological agents used in RA modulate the expression of organokines and have their effects counterbalanced by their secretion and action. Considering that many immunobiological agents are already being used, while others are still under development, we believe that a better understanding of how these drugs alter or interfere with the organokine secretion profile would be a fertile field of research. Indeed, it would also be interesting to study how herbal medicines can exert modulatory effects on the secretion of organokines related to RA and could be used as antirheumatic drugs. Herbal products such as curcumin, for example, have been shown to be a very effective therapy against RA inflammation.

## 5. Conclusions

RA has a significant impact on orthopedic clinical practice worldwide. Early diagnosis and initiation of appropriate management are crucial, as RA patients can develop chronic, erosive arthritis if left untreated or if treatment is delayed. The diagnosis of this disease is essentially a clinical diagnosis; however, radiographic and laboratory investigations provide complementary diagnostic and prognostic information about the condition. In this sense, a better understanding of the biochemical and inflammatory factors involved is necessary.

Organokines are increasingly recognized as critical mediators of disease development, prevention, and control, particularly due to their role in inter-organ and systemic communication and coordination. Much still needs to be done to transpose (translational medicine) and incorporate these data into clinical practice whether for diagnosis, follow-up, or treatment.

Research with organokines in autoimmune chronic inflammatory diseases is a promising direction for future research initiatives. These soluble factors produced by different organs have the potential to be used as adjuvant biomarkers to predict clinical outcomes and propitiate personalized therapy programs. Large-scale studies in RA patients and possibly the use of animal models will be essential resources to provide an enabling environment to pursue future questions that interlink organokines with RA.

## Figures and Tables

**Table 1:** Main characteristics of organokines with simultaneous classification involved in the pathophysiology of RA.

Release	Organokine	Expression in RA	General Functions	Role in RA	References
Hepatokine and adipokine.	Fibrinogen-like protein 1 (FGL1) (HEPASSOCIN)	↑, 10-times higher compared to healthy individuals.	Mitogenic to human hepatocytes (↑ liver regeneration); Rescue role in hyperglycemic crisis; Liver protector; Lipogenic (↑ lipogenesis); ↑ Preadipocyte proliferation; Immune activation; Probable role in cancer development; ↑ IR, probable role in NAFLD.	Predictor of disease activity, progression, and prognosis; Probable role in inhibiting autoimmunity under up-regulated circulation (major inhibition of LAG-3);	[26,139,140,156]
Adipokine and hepatokine.	Angiopoietin-like 4 (ANGPTL-4)	↑, principally in the synovial fluid, bones, and cartilages.	Regulator of plasma triglycerides metabolism by inhibiting the lipoprotein lipase, principally under fasting conditions; Prevents excessive lipid uptake by the heart and skeletal muscles.	↑ Production of MMP-1 and MMP-3 by chondrocytes (↑ cartilage matrix degradation and catabolism); In inflammation-derived hypoxia, and ANGPTL-4 stimulates osteoclasts to bone resorption (principally in elevated RANKL levels); Probably promotes angiogenesis (inhibiting endothelial apoptosis and stimulating endothelial cell migration); ↑ Cardiovascular risk among RA patients, principally due to impaired glucose metabolism.	[113,114,144,145]
Adipokine and hepatokine.	Fetuin-A	↑ or ↓ compared to controls depending on the population.	↑ Insulin resistance in the liver and skeletal muscles; Pro-adipogenic and suppressor of adiponectin secretion and effects.	It may induce pro-inflammatory cytokines production in adipocytes and macrophages involved in RA structures.	[11,147,157,158,159]
Adipokine and hepatokine.	Lipocalin 2 (LCN2)	↑, compared to controls.	Inflammatory biomarker; Role in apoptosis, cell differentiation, fatty acid oxidation, iron transport, inflammation regulation, immune response, and metabolism in general.	Serum levels of LCN2 can be used to indicate structural damage, such as erosions in the early stage of the disease, but cannot be used to monitor disease activity and are not directly related to inflammatory activation; LCN2 can also play a role in the degeneration of joints in RA and could be a biomarker of cartilage degradation in arthritic diseases.	[148,149]
Adipokine and myokine.	Follistatin-like 1 (FSTL1)	↑, compared to controls.	Involved in fatty acid and glucose oxidation; Involved in cardiomyocyte hypertrophy, cardiomyocyte apoptosis, and vascular smooth muscle cell proliferation and migration; It can influence endothelial cells’ survival, migration, and differentiation; It can be involved in tumor growth and metastasis; In inflammatory diseases, it can augment pro-inflammatory cytokines secretion.	Higher levels reflect articular degradation and inflammation in RA; Osteoclastogenic via RANKL-mediated NF-κB activation and M-CSF-induced precursor proliferation; Osteogenic suppressor by ↓ osteoblast differentiation of BMSCs during inflammation; Probably involved in RA fibroblast-like synoviocytes proliferation, migration, invasion, and inflammation; ↑ RA progression by up-regulating IFN-γ signaling pathways and bridging innate and adaptive immune responses; ↑ MMP-1, MMP-3, and MMP-13 gene expression via MAPK, JAK/STAT3, and NF-κB signaling pathways.	[127,128,150,152,153,154,155,160,161]

RA, rheumatoid arthritis; FGL1, fibrinogen-like protein 1; ↑, increase; ↓, decrease; IR, insulin resistance; NAFLD, non-alcoholic fatty liver disease; LAG-3, lymphocyte-activation gene 3; MMP-1, metalloproteinase 1; MMP-3, metalloproteinase 3; MMP-13, metalloproteinase 13; RANKL, receptor activator of nuclear factor kappa B ligand; LCN2, lipocalin 2; FSTL, follistatin-like 1; RANKL, receptor activator of NF-κB ligand; NF-κB, nuclear factor kappa b; M-CSF, macrophage colony-stimulating factor; IFN-γ, interferon gamma; MAPK, mitogen-activated protein kinase; JAK/STAT3, Janus kinase/signal transducer and activator of transcription protein; BMSCs, bone marrow mesenchymal cell.

**Table 2:** Review of studies that evaluated the roles of organokines in RA patients.

References	Organokine (S) Evaluated	Study Design	Sample	Evaluations and Interventions in RA	Primary Efficacy Outcome/ Correlation
Cheleschi et al., 2022 [13]	Adiponectin, chemerin, leptin, resistin, and visfatin.	Case-control study. Italy.	50 RA patients (50–67, 15♂ and 35♀), 50 affected by PsA (55–63 y, 22♂ and 28♀), and 50 controls (40–59, 19♂ and 31♀).	Assessment of the relationships between adipokines and microRNAs in the discrimination between RA and PsA.	Leptin and microRNA-140 were increased in serum of PsA compared to RA or controls and therefore can be biomarkers used to discriminate PsA from RA.
Wahba et al., 2021 [15]	Chemerin, apelin, vaspin, and omentin.	Observational cross-sectional study. Egypt.	150 RA patients (60♂ and 90♀, 44.29 ± 9.4 y) + 150 (75♂ and 75♀, 42.07 ± 11.3 y) healthy individuals.	Assessment of the roles of chemerin, apelin, vaspin, and omentin in RA pathophysiology among patients and their genetic variants, named rs17173608, rs2235306 rs2236242, and rs2274907.	Chemerin and vaspin levels were higher in RA patients and associated with clinical and laboratory parameters of the disease. Further, apelin and omentin levels were lower.
Gould et al., 2021 [122]	FGF-21.	Observational and longitudinal study. USA.	113 RA participants aged between 18–70 y were primarily enrolled, and 84 attended the follow-ups.	Assessment of associations between FGF-21 and adverse changes in body composition longitudinally and physical functioning in individuals affected by RA.	FGF-21 levels were positively associated with obesity and secretion of pro-inflammatory cytokines in RA and with worsening physical function in RA.
Baker et al., 2021 [162]	Adiponectin leptin and FGF-21.	Observational cross-sectional study. USA.	419 older-aged patients diagnosed with RA.	Determine associations between adipokines levels and abnormal body composition among patients with RA.	Grater fat mass associated with lower adiponectin and higher FGF-21 serum levels. Adipokines associates with both excessive adiposity and low lean mass in individuals with RA.
Vazquez-Villegas et al., 2021 [163]	Chemerin.	Observational cross-sectional study. Mexico.	82 women diagnosed with RA (43 with functional disabilities and 39 without functional disabilities, 35–77 y and 30–79 y, respectively).	Elucidate if chemerin serum levels are associated with functional disabilities among women affected by RA.	Higher chemerin serum levels are significantly associated with functional disabilities among RA women, whereas no other blood biomarkers correlated with loss of function are present.
Gonzalez-Ponce et al., 2021 [164]	Chemerin.	Observational cross-sectional study. Mexico.	210 RA women patients (56.59 ± 11.25 y).	Evaluate whether serum chemerin is a biomarker of disease activity among RA patients.	Higher chemerin serum levels increase the risk of moderate and severe RA disease, supporting chemerin as a joint inflammatory biomarker in RA.
Zhang et al., 2021 [165]	Adiponectin, leptin, resistin, and visfatin.	Case-control cohort study. Sweden;	82 obese individuals that developed RA during follow-up matched with 410 controls + 88 additional matched pairs.	Identity if adiponectin, leptin, resistin, and visfatin serum levels associate with RA risk and if adiponectin performs an association with RA risk that is independent of the other adipokines.	In the included obese individuals, higher levels of adiponectin were associated with increased risk for developing RA but not higher levels of leptin, resistin, or visfatin.
Wahlin et al., 2021 [16]	Osteoprotegerin and osteocalcin.	Prospective cohort study. Sweden.	79 patients newly diagnosed with RA (≤60 y at diagnosis).	Evaluate possible interactions between subclinical atherosclerosis and markers of bone turnover, regulators of bone formation, and BMD in patients with RA.	Subclinical atherosclerosis in individuals on new-onset with RA is associated with osteoprotegerin and osteocalcin but not with markers reflecting ongoing bone turnover or BMD.
Hanzhu Chen et al., 2021 [14]	Adiponectin.	Mendelian randomization study. Multicenter.	14,361 cases and 43,923 controls. Results were analyzed with data from another 11,437 cases and 604,953 controls to decrease bias risk.	Assessment of a possible causal relationship between adiponectin and RA risk.	There was no evidence to support a causal association between adiponectin on RA risk and of RA on circulating levels of adiponectin.
Jiliang Chen et al., 2021 [166]	Leptin.	Cohort analytical study. China.	223 RA patients (68.1 (57.2–75.6 y, 115♂ and 108♀)).	Explore whether leptin is associated with increased cardiovascular risk.	Elevated serum leptin levels were significantly associated with the prediction of cardiovascular events among RA individuals.
Taylan et al., 2021 [28]	Leptin.	Cohort analytical study. Turkey.	47 RA patients with early disease (54 ± 15 y, 13♂ and 34♀) and 25 controls (51 ± 14, 3♂ and 22♀).	Explore the relationship between serum leptin levels and disease activity among RA patients.	Leptin serum levels were significant and directly associated with RA disease activity, and treatment with DMARDs decreased the levels of this adipokine.
Vuolteenaho et al., 2021 [29]	Resistin.	Post hoc analyses of an investigator-initiated, multicenter, controlled study. Finland.	99 early, DMARDs-naïve RA patients.	Investigation of whether resistin could be associated with not only inflammation but also disease activity among DMARDs-naïve RA patients as well as disease progression.	Serum pretreatment resistin levels were associated with an increased risk of erosive disease in the early stages of RA disease.
Liu et al., 2020 [17]	Fibrinogen-like protein 1 (FGL1) (HEPASSOCIN).	Large-scale cohort two-centered study. China.	1244 participants divided into 5 cohorts: #1 had 35 RAP with MTHDA + 60 healthy; #2 had 38 RAP with MTHDA + 15 RAP in remission/with LDA + 28 healthy; #3 had 221 RAP with MTHDA + 84 RAP with LDA + 102 RAP in remission, + 47 RAP with MTHDA, and 233 healthy; #4 had 82 RAP with MTHDA before DMARD treatment + 23 RAP with MTHDA + 26 RAP with LDA + 33 RA in remission after DMARD intervention; and #5 had 35 healthy individuals + 23 RAP in remission + 24 RAP with low to high disease activity + patients without RAP.	Evaluation of biomarkers that can precisely indicate and monitor RA disease activity and provide adequate therapeutics.	FGL1 could positively predict RA disease activity and its prognosis. Clinically, FGL1 was considered useful for predicting RA progression.
Qu et al., 2020 [18]	Sex hormone-binding globulin (SHBG)	Mendelian randomization study. China.	In all, data from 28,837 European ancestry individuals were assessed. This study also analyzed Mendelian association of SHBG and osteoarthritis and ankylosing spondylitis.	Determine whether the concentrations of SHBG are correlated with the elevated risk for developing RA.	Positive causal effects of circulating SHBG levels were found to determine RA development risk with no sex specifications.
Murillo-Saich et al., 2021 [19]	Myostatin.	Cross-sectional study. Mexico.	127 women were enrolled without any rheumatological diagnosis (24–85 y) and 84 RA women (24–89 y).	Evaluate the association between serum myostatin levels and inflammatory parameters among RA women.	Myostatin associated significantly with RA disease activity by augmented inflammatory biomarkers, suggesting myostatin roles in muscle wasting and inflammation among RA women.
Gamez-Nava et al., 2022 [139]	Irisin.	Cross-sectional study. Mexico.	148 women with RA (≥45 y) and 97 control women.	Evaluate the association between serum irisin levels and osteoporotic vertebral fracture among RA diseased women.	Decreased levels of irisin associated significantly with the occurrence of osteoporotic vertebral fracture.
Gamal et al., 2020 [167]	Irisin.	Cross-sectional study. Egypt.	58 RA patients (44.12 ± 11.78 y) and 30 matched controls.	Evaluate if irisin levels were correlated with good sleep quality in RA individuals compared to controls.	Irisin levels were decreased in RA patients with poor sleep quality compared to controls.
Soliman et al., 2022 [156]	Irisin.	Cross-sectional study. Egypt.	60 RA patients (47.03 ± 9.5 y) and 30 healthy individuals.	Asses if serum irisin levels in RA patients correlate significantly with cardiovascular risk factors and subclinical atherosclerosis occurrence.	Decreases in irisin serum levels correlated significantly with increased cardiovascular risk factors occurrence and subclinical atherosclerosis prediction.

RA, rheumatoid arthritis; RAP, rheumatoid arthritis patients; PsA, psoriatic arthritis; MTHDA, moderate to high disease activity; FGL1, fibrinogen-like protein 1; DMARD, disease-modifying anti-rheumatic drugs; ↑, increase; ↓, decrease; ♂, man/men; ♀, woman/woman; y, year/years; FGF-21, fibroblast growth factor 21; GWAS, genome-wide association studies; DMARD, disease-modifying anti-rheumatic drug; BMD, bone mineral density; SHBG, sexual hormone-binding globulin.

## Data Availability

Not applicable.
